# Azobenzene-Based Solar Thermal Fuels: A Review

**DOI:** 10.1007/s40820-022-00876-8

**Published:** 2022-06-29

**Authors:** Bo Zhang, Yiyu Feng, Wei Feng

**Affiliations:** 1 School of Materials Science and Engineering, Tianjin University, Tianjin, 300350 People’s Republic of China; 2 Tianjin Key Laboratory of Composite and Functional Materials, Tianjin, 300350 People’s Republic of China; 3 Key Laboratory of Materials Processing and Mold, Ministry of Education, Zhengzhou University, Zhengzhou, 450002 People’s Republic of China

**Keywords:** Azobenzene, Solar thermal fuels, Nanocarbon template, Controllable energy release, Phase change materials

## Abstract

The energy storage mechanism of azobenzene is based on the transformation of molecular cis and trans isomerization, while NBD/QC, DHA/VHF, and fulvalene dimetal complexes realize the energy storage function by changing the molecular structure. Acting as “molecular batteries,” they can exhibit excellent charging and discharging behavior by converting between *trans* and *cis* isomers or changing molecular structure upon absorption of ultraviolet light. Key properties determining the performance of STFs are stored energy, energy density, half-life, and solar energy conversion efficiency. This review is aiming to provide a comprehensive and authoritative overview on the recent advancements of azobenzene molecular photoswitch system in STFs fields, including derivatives and carbon nano-templates, which is emphasized for its attractive performance. Although the energy storage performance of Azo-STFs has already reached the level of commercial lithium batteries, the cycling capability and controllable release of energy still need to be further explored. For this, some potential solutions to the cycle performance are proposed, and the methods of azobenzene controllable energy release are summarized. Moreover, energy stored by STFs can be released in the form of mechanical energy, which in turn can also promote the release of thermal energy from STFs, implying that there could be a relationship between mechanical and thermal energy in Azo-STFs, providing a potential direction for further research on Azo-STFs.

## Highlights

- The strategy to achieve alternate mixing exotherm through metal–organic frameworks material that may be proposed to solve the potential drawbacks of azobenzene with nanocarbon template is discussed.
- Factors that can affect the isomerization of the Azo unit include but are not limited to light, heat, catalysts, solvents, electric field, magnetic field, force were discussed to achieve controllable azobenzene energy release.

## Graphical abstract

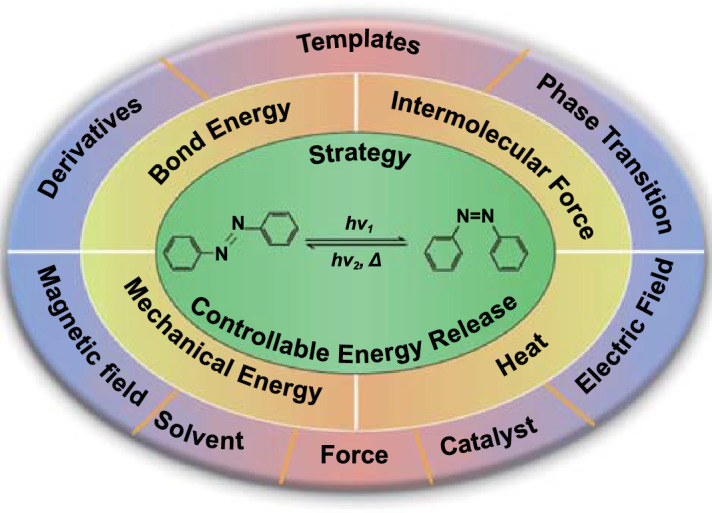

## Introduction

The industrial revolution has promoted the development of human civilization at the expense of a large amount of non-renewable fossil energy resources and gave rise to some serious problems, such as energy shortages and environmental pollution, which were gradually realized since the beginning of the twenty-first century [1]. Renewable fuels are actively sought as substitutes for fossil fuels to reduce pollutant emissions and transition to a clean and sustainable new energy field, which is led by solar and wind energy [2, 3].

**Fig. 1:**
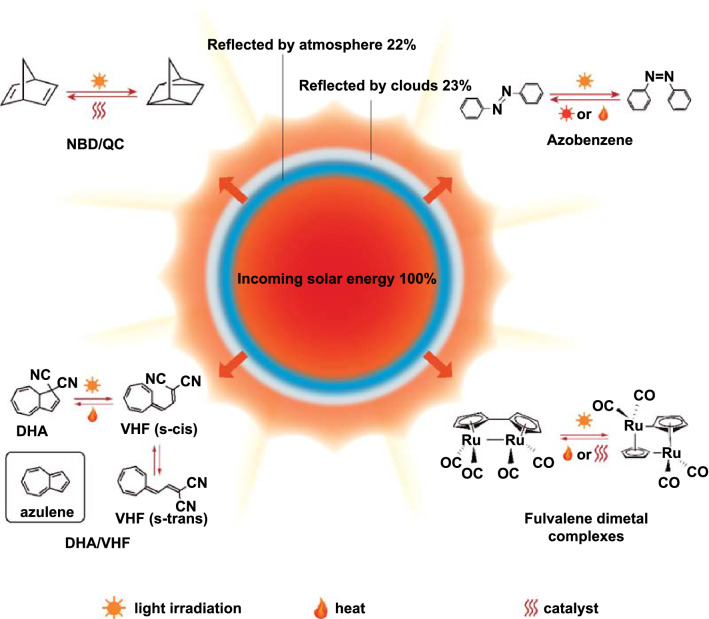
Photoisomerization of four different types of molecular photoswitches [10]

According to statistics, worldwide primary energy consumption in 2001 was 425 × 10^18^ J, which is the amount of energy released by the sun in just one hour [4, 5]. Compared with nuclear, wind, and tidal energy, solar energy has a lower threshold and abundant supply, which gradually made solar energy a research hotspot [6]. Energy produced by the sun blackbody radiation can be divided into three parts: ultraviolet (UV) light, visible light, and infrared light. In fact, most UV fails to penetrate the atmosphere and reach the earth’s surface, making visible light and infrared light the main components of the sunlight (Fig. 1) [7, 8]. As shorter wavelength UV has higher energy than visible and infrared light, it is more suitable for the stimulation of electronic energy level transitions. Therefore, UV has gradually become a major focus of studies on solar energy. Solar energy also has some obvious shortcomings. The collection efficiency of solar energy depends significantly on the time of the day and weather conditions. To industrialize solar energy, the development of high-efficiency solar energy collection devices and stable solar energy release devices is required, which has become a current research hotspot [9]. Current solar heat conversion devices can be mainly divided into two types: sensible heat energy storage devices and latent heat energy storage devices.

Sensible heat storage is the only commercially viable method of solar thermal energy storage because of its energy storage density and mature stage of research. In comparison, latent heat storage devices are a potential new generation research hotspot because they possess the following advantages: stronger temperature control ability, smaller size, and simpler structure [11–13].

### Solar Thermal Fuels

As a class of latent heat energy storage materials, solar thermal fuels (STFs) can store the collected solar energy in their chemical bonds and release energy in the form of thermal energy under specific conditions. Their characteristics provide a possibility for solar energy stable output [9, 14–16]. For the better use of solar energy, the spectral range that excites STFs should be close to the sunlight spectrum. The content of the specific sunlight wavelength in the sunlight spectrum that meets the conditions for exciting STFs should also be focused, which means that the excitation range of STFs should be red-shifted to the visible light region, if possible. STFs must possess chemical bonds with high photochemical activity, such as conjugated double bonds and N=N.

The measurement standards for STFs have been formulated to make full use of solar energy: energy density (ED), power density (PD), energy storage half-life, and solar energy conversion efficiency. To effectively use solar energy and meet the industrialization needs of solar energy, STFs need to have high ED and PD, long half-life, high solar energy conversion efficiency, high photothermal cycling stability and low cost [16, 17]. Based on the above conditions, the attention gradually turned to azobenzene, NBD/QC, DHA/VHF, fulvalene, and others [16, 18].

Under UV radiation, norbornadiene (NBD) is converted to quadricyclane (QC). With the help of a catalyst, QC can be converted back to NBD and release a large amount of energy (∆*H* = 0.97 MJ kg^−1^, as shown in Fig. 1) [14]. However, the NBD energy storage system still has many problems, such as poor solar spectrum matching and low quantum yield, causing the lower solar energy utilization rate [19]. Therefore, red-shifting the NBD’s excitation light wavelength through molecular modification and other means is imperative [20, 21]. Earlier, it was discovered that the expansion effect of the conjugated *π* system caused by the diaryl substitution group can cause a strong red shift in the NBD/QC system. Mikael et al. [22] used single-reference and multireference methods (complete active space techniques) to model the reverse conversion absorption spectra, potential energy storage, and thermal barrier of diaryl-substituted NBDs. They confirmed two possible reaction paths for the thermal reverse conversion of quadricyclanes and found that the absorption spectrum depends critically on the *π*-coupling angle of the aryl ring. Currently, researchers began to use donor and acceptor groups with lower molecular weight (–CN, –F) to obtain NBD derivatives with higher ED and absorption spectra better matched with the solar spectrum. At the same time, it is worth mentioning that although pure liquid NBD possesses a higher energy storage density, special solvents can also play a role in enhancing the utilization of sunlight [23]. QC needs catalysts to be reversed to NBD, and the isomerization process can generate 89 kJ/mol of energy [24]. Currently, there are three main excitation methods: (1) homogeneous transition metal catalysts or Lewis acids in solution, (2) on the surface of heterogeneous catalysts, and (3) electrochemical catalysis [25]. Generally speaking, the solution reaction, in which side reactions like polymerization may occur, is less controllable. Side reactions also reduce the energy storage density of the NBD/QC system [26]. Therefore, in recent years, heterogeneous catalyst and electrochemical catalysis have attracted increasing attention. Currently, the most popular heterogeneous catalysts are Ni-based and Pt-based surface catalysts. It is also worth mentioning that the sensible heat storage technology can increase the NBD/QC system efficiency to 80% without significantly decreasing the efficiency of the sensible solar water heating system [27, 28].

Dihydroazulene (DHA) differs from NBD in that its conversion occurs in two steps. When irradiated with UV light, DHA first undergoes a ring-opening reaction and is converted into *cis*-vinylheptafulvene (s-*cis*-VHF). Subsequently, s-*cis*-VHF is further converted into *trans*-vinylheptafulvene (s-*trans*-VHF), which is more stable, forming a dynamic equilibrium with it (as shown in Fig. 1). Compared with the minimum UV light wavelength of 250 nm required to initiate the original NBD/QC and the light-induced competition phenomenon, the solar energy utilization efficiency of this system is higher, and the quantum yield can reach 0.55 [29]. In fact, the DHA molecule itself has multiple carbon sites for molecular modification, making it easy to functionalize and has a high structure tunability, energy storage, and release, which is unmatched by the NBD system [30–33]. The highest energy storage density of the molecularly modified DHA/VHF derivatives system can reach 0.73 kJ kg^−1^, which almost equals the energy storage density of the original NBD/QC system (≈ 0.7 kJ kg^−1^) [34]. Its molecular modification strategy is similar to NBD: By inserting different functional groups at different positions of the DHA or VHF molecule, its half-life and energy storage density can be significantly affected. At present, the DHA molecule is mainly modified in two directions. The first is the removal of a cyano group on C1. Because the reverse reaction is insignificant, researchers have discovered that the energy storage density of the monocyano DHA derivatives with one cyano group removed from the C1 molecule can be doubled (0.25 MJ kg^−1^) [35]. The second direction of modification is the addition of donor–acceptor groups such as benzo ring and benzene ring. The forces of the groups headed by the benzo ring (conjugation, induction, etc.) and their locations have a great impact on the energy storage density and stability of the DHA/VHF system. It is worth noting that the DHA molecule has a strong polar substitution group (–CN), which makes the solvents have a greater impact on the properties of the DHA/VHF energy storage system than on those of the NBD/QC energy storage system [30, 32, 36].The DHA/VHF system has unique applications like photochromic units and STFs, which are expected to become tools for the collection and storage of solar energy.

The fulvalene dimetal complex system undergoes the cleavage and reformation of X–X and C–X bonds (X is Ru, Fe, Os, and other metal atoms) under UV light (350 nm) and stores the photon energy in a metastable state. Energy can be thermally reversed or catalytically released by conformational isomers. The energy storage density of the fulvalene dimetal complex system is 0.11–0.19 MJ kg^−1^ (Fig. 1). However, because of low energy storage density and high cost, it is more seldom researched and applied than alternatives [37–46].

Azobenzene has two configurations: *cis* and *trans*. *Trans*-azobenzene has relatively stable properties. It undergoes a *π* → π* transition after being excited by UV light. Most azobenzene can be isomerized from the *trans* configuration to the *cis* configuration by UV irradiation, while a few molecules may be reduced to *trans* isomers. *Cis*-azobenzene is converted back to *trans*-azobenzene with the emission of heat through heating or visible light irradiation.

However, the high activation energy of covalent bonds requires irradiation with UV, which means their solar utilization efficiency is not ideal. Based on the problems outlined above, improvement in their application value requires modifying the molecules to adjust the absorbed wavelength spectrum to the natural solar spectrum [47].

### Advantages of Azobenzene-Based Solar Thermal Fuels

Azobenzene has its own chromophore group, and its *cis*–*trans* isomerization process will be accompanied by color changes. Therefore, compared with other STFs energy storage systems, researchers can better investigate the energy storage process of Azo-STFs without adding additional chromophores.

Also, azobenzene molecules undergo isomerization under light irradiation, which can cause a small change in molecular size (the molecular axis changes from 9 to 5.5 Å). The molecular size changes caused by the isomerization of azobenzene molecules can be superimposed on each other, which results in visible macroscopic deformation. Currently, light-driven flexible actuators based on azobenzene photo-responsive polymers have attracted the attention of the scientific community because of their fast, flexible, and reversible light deformation conversion capabilities, from solar thermal storage to molecular motors, memory, and manipulators [48–50]. A variety of polymer structures, including main chain polymers, side-chain polymers, cross-linked networks, and block copolymers, have already been designed and prepared to attain light-driven conversion through the force between Azo unit and polymer [51, 52]. This shows that the controlled release of the energy stored by azobenzene in the form of mechanical energy can be realized.

Although the energy storage density of azobenzene is not as high as that of the NBD/QC derivative system (up to 0.97 MJ kg^−1^), unique isomerization mechanisms during the heat storage and release processes endow azobenzene energy storage system with more diverse application prospects which can be used in many fields such as STFs, light-driven actuators, data storage, seawater desalination, and deep space probes [16, 53–59].

## Advances in Azobenzene Materials for Solar Thermal Fuels

**Fig. 2:**
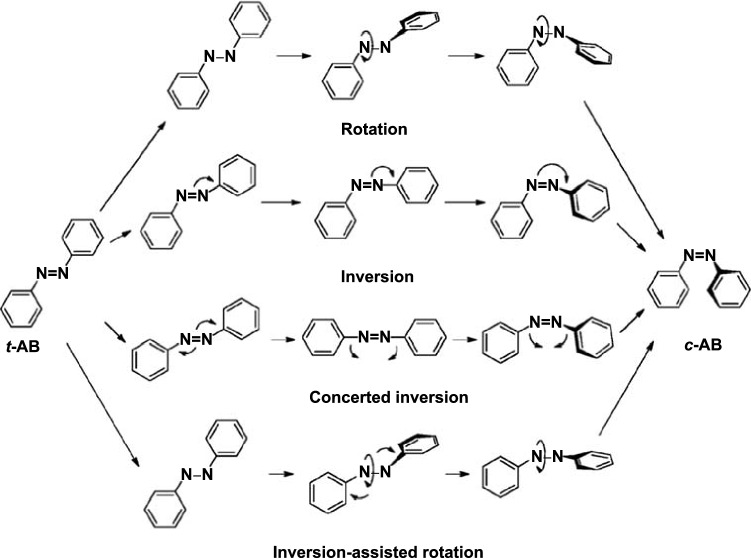
Four known mechanisms for azobenzene *trans* to *cis* isomerization [60]

There are four known mechanisms for azobenzene *trans* to *cis* isomerization: (1) the rotation mechanism wherein a 180° rotation takes place around the N=N double bond; (2) the inversion mechanism, which proceeds via a dipolar transition state. Different azobenzene derivatives have different isomerization mechanisms; (3) the concerted inversion, both N=N–C bond angles increase to 180° generating a linear transition state and (4) the inversion-assisted rotation, large changes in the C–N=N–C dihedral angle and smaller but significant changes in the N=N–C angles occur simultaneously [60]. The transition state formed in concerted inversion has no net dipole moment, whereas the other three pathways possess polar transition states. Relaxation from all four transition states can afford either the *cis* or the *trans* isomer; therefore, all four mechanisms predict photostationary states consisting of both isomers (Fig. 2).

Studies suggest that substitution on the phenyl rings and the environment determine the pathway taken. In unsubstituted, neutrally substituted, and sterically constrained azobenzene, the thermal *cis → trans* isomerization takes place via the inversion mechanism. Interestingly, for push–pull azobenzene, the reaction mechanism changes from inversion in nonpolar solvents to rotation in polar solvents [61–64].

The B3LYP/6-31G* model and Eyring transition state theory had been chosen by Dokić et al. to study around 90 azobenzene derivatives’ *cis → trans* isomerization in the gas phase and in a polarizable environment [63]. They found that in most cases, isomerization proceeds through a linear transition state, which is indicative of an inversion mechanism. The transition state, however, is not reached by pure inversion along the angle *α* but rather by simultaneous rotation around the C–N=N–C dihedral angle *ω*. The linear transition state can be stabilized very efficiently by acceptors in ortho and para positions. Three years later, Robertus et al. reported a photochemical and kinetic study on the thermal relaxation reaction of a double azobenzene system, in which two azobenzene photochromic units are connected via a phenyl ring [65]. Their results indicated that the thermal *cis* to *trans* isomerization of one of the switching units does not significantly affect the second unit. Their study proved that the isomerization of the azo unit has a certain degree of independence, which provides a theoretical basis for linking the azo unit to other molecules.

A more interesting phenomenon is worth noting here: The external environment has a greater impact on the isomerization of azobenzene and its derivatives than on that of several photothermal chemical energy storage materials introduced earlier. It means that the release of energy stored during the isomerization of azobenzene through various means can be controlled, which is discussed in Sect. 3 of this paper.

The half-life of the whole process is 4.2 d (the principle is shown in Fig. 1) [66–68]. In 1978, Adamson et al. [69] measured the enthalpy change of the reaction (0.02 MJ kg^−1^). It was found that the energy storage density of azobenzene can be further increased to 0.54 MJ kg^−1^ through molecular modification and other methods. In addition, the energy storage density of solid azobenzene is generally greater than that of liquid azobenzene. The larger energy storage density and lower cost made azobenzene a research hotspot. However, azobenzene also has some problems that cannot be ignored.

In 1983, Olmsted et al. [70] found that the thermal transition rate of the azobenzene system is faster and solubility in polar solvents is lower than previously considered. In addition, because of the molecular properties, the system can only be used in a high-temperature environment, and its low-temperature tolerance is poor. In addition, UV light can trigger a small amount of *cis*-azobenzene while initiating the reaction of *trans*-azobenzene, meaning that *cis*- and *trans*-azobenzene compete for light, which inevitably reduces the utilization of solar energy.

There are basically two approaches to solve the above problems, namely, molecular modification and template method. Molecular modification can effectively red-shift the UV light excitation range and increase the solubility of azobenzene while the template method can extend the half-life in most instances. In addition, mixing different azobenzene derivatives into “full-spectrum” Azo-STFs can also improve solar energy utilization.

### Azobenzene Derivatives

The excitation range of azobenzene can be red-shifted to the visible light region using molecular modification and other means. In recent years, to enhance the Azo-STFs’ performance, three ways were mainly explored: (1) adopting a non-solvent azobenzene energy storage structure; (2) combining the exothermic azobenzene photoisomerization with the exothermic phase change to increase its energy storage density; and (3) using molecular interaction forces (such as hydrogen bonds, van der Waals forces) between the azobenzene derivative molecules to increase the energy storage density of the azobenzene system.

It is worth noting that to enhance the energy storage performance of azobenzene, combining it with the phase-change mechanism is one of the popular strategies [71]. Phase change materials (PCMs) can provide a stable temperature span during the release of stored latent heat [72]. PCMs can be divided into two categories: inorganic and organic PCMs. Inorganic PCMs have several fatal problems, such as overcooling and phase separation, which greatly limit the application scope of inorganic PCMs. The organic PCMs like paraffin and sugar alcohol can avoid some of the fatal problems of inorganic PCMs [73–76]. The introduction of the phase-change mechanism can endow azobenzene materials with properties, such as high energy storage density and wide melting temperature range [77, 78]. In recent years, the theory of room-temperature photochemical crystal-liquid transition (PCLT) has been continuously improved for azobenzene optical switches. The whole process is roughly as follows: Azo molecules capture photons to perform *trans* → *cis* photoisomerization and spontaneously absorb heat from the surrounding environment when they overcome the molecular stacking interaction of the crystal → liquid phase transition. In the process of *cis*-liquid phase → *trans* crystal transition (by external triggers, such as light irradiation or heating), through the *cis* → *trans* isomerization and the crystallization of *trans* isomers in the liquid phase, the stored energy can be released in the form of chemical and crystallization enthalpy (Δ*H*_isom_ and Δ*H*_cryst_). This process enables simultaneous storage of photon energy and environmental heat and their release as high-temperature heat on demand.

**Fig. 3:**
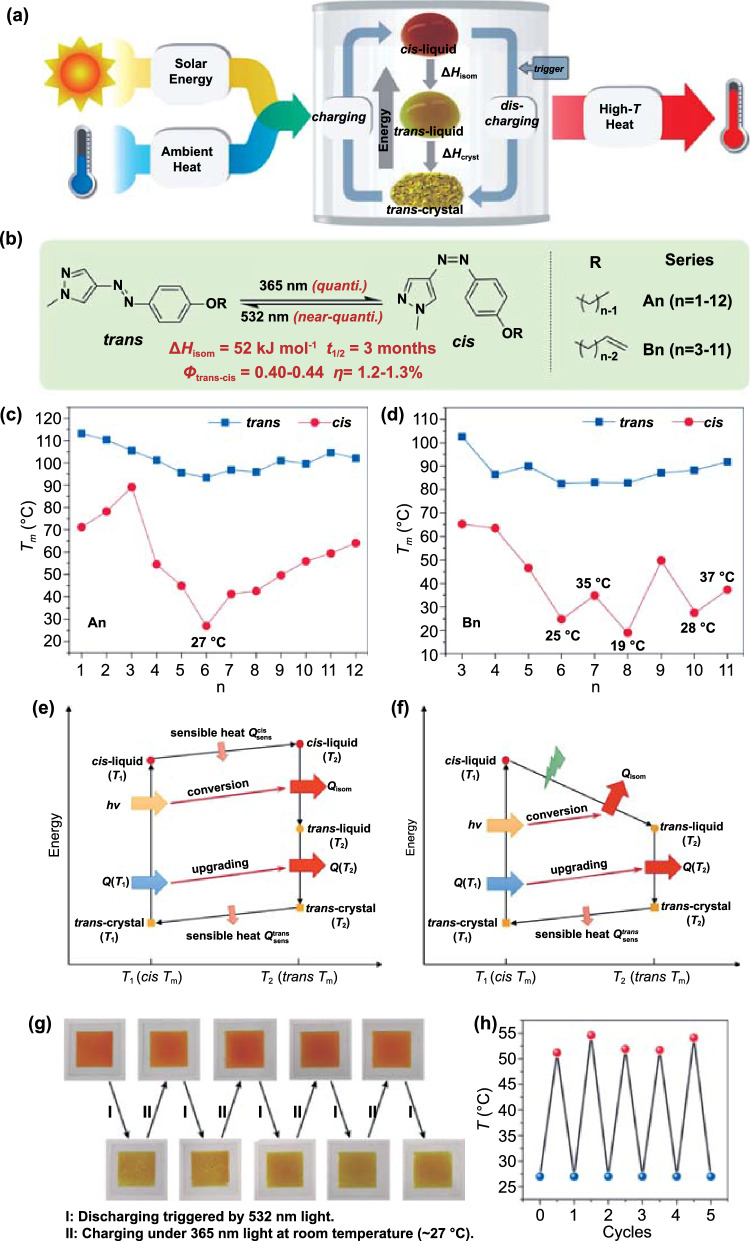
**a** An illustration of a molecular solar thermal battery that is charged with solar energy and ambient heat and releases high-T heat using PCLTs of Azo switches. The two forms of energy are simultaneously harvested/stored by *trans*-crystal → *cis*-liquid transition, and during discharge, the *cis*-liquid releases two streams of heat (Δ*H*_isom_ and Δ*H*_cryst_) by *cis* → *trans* isomerization in the liquid phase and crystallization of *trans* isomers. **b** Photochemical properties of pzAzo ethers and two series of derivatives, *A*_*n*_ and *B*_*n*_. **c**, **d** *T*_m_ of *A*_*n*_ and *B*_*n*_ in two isomeric forms as a function of chain length, respectively. All *T*_m_ were determined by differential scanning calorimetry (DSC) except that of *cis*-B8, which was measured by the Thiele tube method. Ideal thermodynamic cycles where energy release is triggered by **e** heating to *trans* *T*_m_ and **f** visible light irradiation. During a full physicochemical cycle, light energy (*hγ*) is converted to isomerization heat (*Q*_isom_), and the low-T heat (*Q*(*T*_1_)) is converted to high-T heat (*Q*(*T*_2_)). **g** Photographs of the film in alternate charged and discharged states. **h** The temperature lift during five charge/discharge cycles. Only the peak *T*_max_ (red dots) and ambient temperature (blue dots) are shown [79]

The synergistic effect of the two forms of energy enables the environmental heat collected in the liquid of low-melting point metastable isomers during photochemical melting to be released as high-temperature heat through the recrystallization of the high-melting point parent isomers, and the energy capacity they provide is higher than that of traditional solar/thermal energy storage systems based solely on molecular photoisomerization or phase change. The azo-molecule switch designed by Zhang et al. can achieve a high weight–energy density (0.3–0.4 MJ kg^−1^) and has long-term storage stability (3 months of *cis* isomer half-life at 25 °C). When applied in rechargeable thin-film solar thermal batteries, it can provide a weight–power density of approximately 2.7 kW kg^−1^ after being triggered by 365-nm LED light at room temperature and increase the temperature in the surrounding environment by more than 20 °C [79] (Fig. 3). Zhang et al. found that in the PCLT cycle, *T*_m_ can be decreased by forward photoisomerization and increased by the reverse isomerization. Moreover, problems such as the low ED and high temperature rise that stem from the properties of azobenzene are still unresolved. In fact, most Azo-STFs need to be heated above 60 °C to release the stored energy. For this reason, the maximum temperature difference between the states before and after the energy release is limited to 2–15 °C, which is not economical for practical applications.

**Fig. 4:**
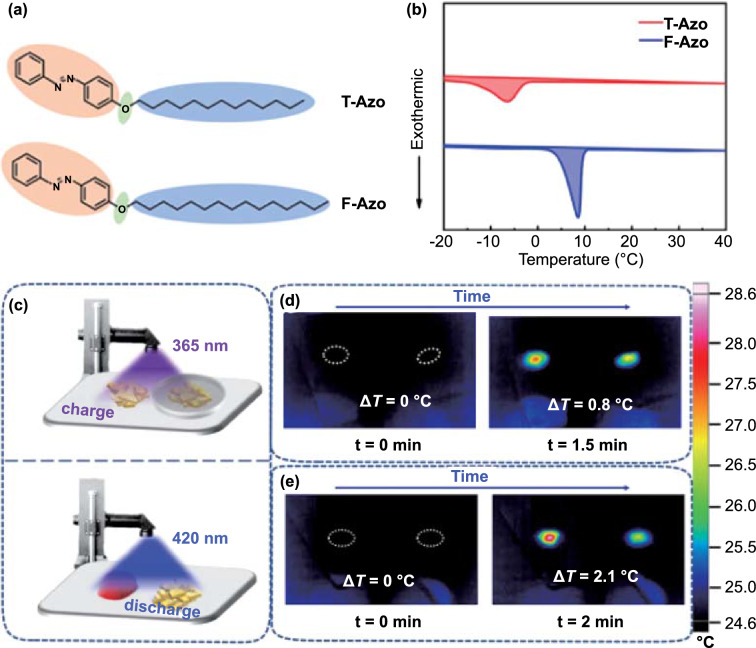
**a** Chemical structure of T-Azo and F-Azo (the red part represents the isomerization unit, the green part represents flexible linker, and the blue part represents the phase change unit). **b** DSC traces of charged T-Azo (red line) and F-Azo (blue line) measured by cooling from 40 to − 20 °C, **c** Schematic of energy storage under UV light (365 nm) irradiation and release at 25 °C induced by blue light (420 nm). IR thermal image of the charged **d** T-Azo and **e** F-Azo irradiated with blue light [86]. (Color figure online)

It was found that if the melting point (*T*_m_) and crystallization temperature (*T*_c_) of the *trans*- and *cis*-azobenzene compounds are higher and lower than room temperature, respectively, light may cause a reversible solid–liquid transition, which can not only improve the heat storage capacity but also provide a theoretical basis for the realization of low-temperature heat release, implying that the preparation of Azo compounds with suitable *T*_c_ or *T*_m_ before and after photoisomerization is the key to solving the problem of low-temperature exotherm [80–85]. Based on this consideration, Feng et al. [86] investigated solar energy utilization of long-chain Azo compounds (T-Azo, F-Azo), which integrate photoinduced solid–liquid transformation and photoisomerization functions, at low temperature (Fig. 4). By optimizing the length of the alkyl chain, the *T*_c_ and *T*_m_ of the *cis*-rich azobenzene can be adjusted to a suitable temperature range, which lays a solid foundation for the control of *T*_c_ and *T*_m_ through molecular engineering. Light-controlled heat release was realized at a relatively low temperature, which provides unique data suggesting that Azo compounds with photoinduced solid–liquid transition and photoisomerization characteristics may overcome the challenge of high heat release temperature in Azo-STFs.

**Fig. 5:**
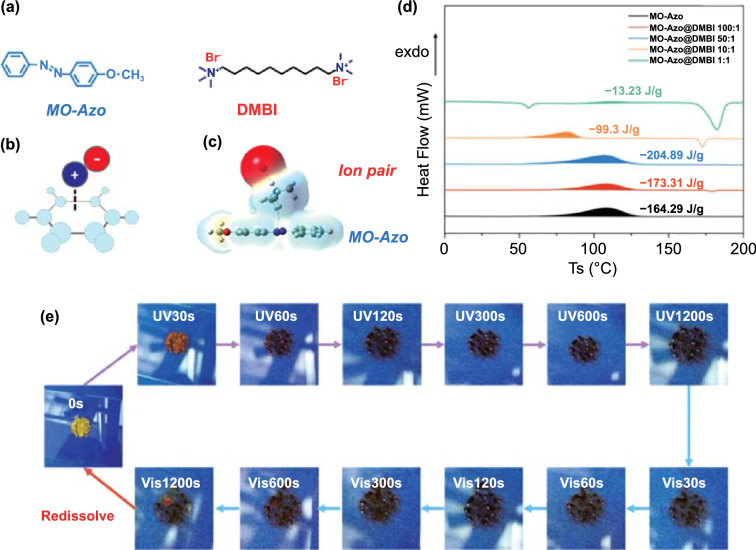
**a** Materials used in the Cation–*π* interaction for enhancement of Azo-STF, **b** Scheme of “Cation–*π*” interaction, **c** Simulation result of “Cation–*π*” interaction **d** Energy storage/release property and stability of. DSC results of MO-Azo@DMBI X:1 with different DMBI content (“MO-Azo” corresponds to 4-methoxyazobenzene, “DMBI” is the abbreviation for decanoquaternary amine bromide, and “X:1” is the mole ratio of these two compounds, respectively); **e** Real-time photographs of MO-Azo@DMBI 50:1 during one “charging–discharging” cycle [90]

Phase transition is also a macroscopic manifestation of intermolecular forces. However, it is worth noting that some intermolecular forces such as *π*–*π* stacking may lead to high molar extinction coefficients of chromophores, which in turn impair the energy storage performance of Azo-STFs. In the meantime, weak interactions, such as the London dispersion as part of the Van der Waals interaction which can loosen the connections between molecules, may be a good choice for adding beneficial stabilizing effects to the STFs system [87, 88]. Anne Kunz et al. [89] demonstrated that attractive London dispersion interactions between molecules stabilize the *trans* isomer in bis-azobenzene connected by different alkyl bridges. DSC measurements showed that the energy stored by each azo unit can be increased to more than 3 kcal mol^−1^ with the help of the interaction, compared with the parent azobenzene. In recent years, in order to further explore the effect of variable types of intermolecular forces on the Azo-based STFs, Yu [90] introduced a “cation–*π*” interaction into an Azo-STFs system, achieving 24.7% (164.3 to 204.9 J g^−1^) enhancement and good stability in energy storage density (Fig. 5).The competition mechanism of the “cation–*π*” interaction and “*π*–*π* stacking” could not only weaken the energy barrier, but also decrease the high molar extinction efficiency of Azo-STFs, demonstrating the great influence of aromatic groups on the Azo-STFs system.

**Fig. 6:**
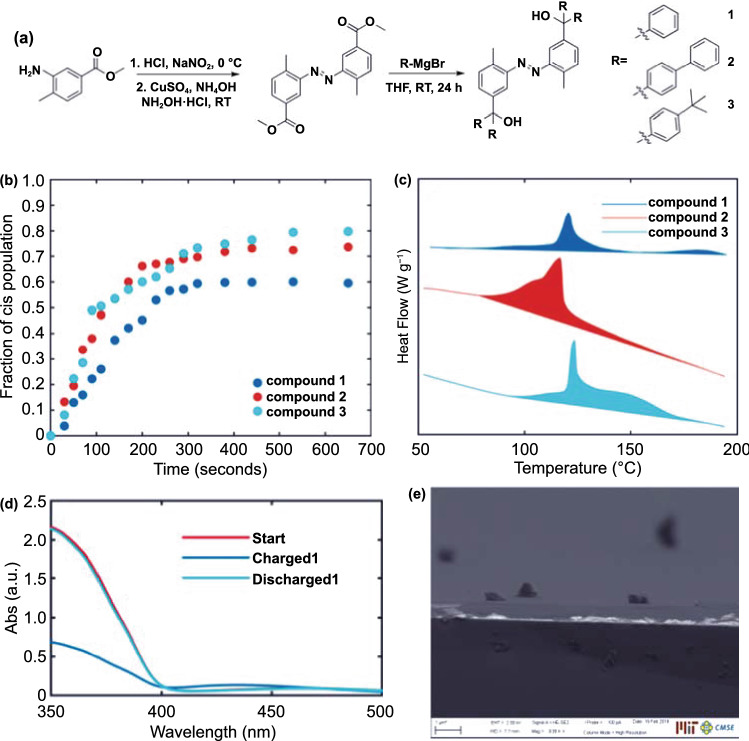
**a** Schematic of synthesis of compounds 1–3 via the diazotization of an aniline precursor followed by the addition of different aryl groups using Grignard reagents. **b** Chargeability of compounds 1, 2, and 3. The process was done ex situ, where the sample was charged under 365 nm UV light for a set time, and then solid-state UV–Vis spectra were obtained. **c** DSC traces for compounds 1, 2, and 3 from top to bottom. The y-direction represents heat release, and the region enclosed in the flat baseline represents the area integrated to obtain energy release. **d** UV–Vis spectrum of light charging of compound 3 films and heat discharging from them. **e** Cross-sectional scanning electron microscopy (SEM) of the compound 3 film [59]

In addition, constructing an artificial hindrance between Azo molecules can also weaken the “*π*–*π* stacking” [15]. Grossman et al. designed three azobenzene derivatives functionalized with bulky aromatic groups (phenyl, biphenyl, and tert-butyl phenyl) to demonstrate the possibility for enhancing Azo-STFs by hindering the *π*–*π* stacking. Thermal stability of the film is a key indicator for the thermally triggered STFs. The large functionalization greatly improves the stability of the film (the thermal stability of the solid film 3 increased from approximately 75 to 175 °C). The compound prepared by Cho et al. [59] reported a record high ED for solid STFs (135 J g^−1^), which is an increase of more than 30% compared with that of the previously reported solid azobenzene functionalized polymer. In addition, the rechargeability of the solid state has been improved from 40 to 80% (Fig. 6). Their results demonstrated that breaking the planarity of molecules plays a vital role in the performance of STFs and proved that molecular engineering is an effective way to increase the energy storage, charging capacity, and thermal stability of the STF films. The molecular designs and their performance metrics have been summarized and compared in Table 1.

**Table 1:** Parameters related to azobenzene derivative STFs

Azo-based molecules	Energy density			*t*_1/2_	State	Cycles	Charging degree	References
	kJ mol^−1^	J g^−1^	Wh kg^−1^					
Azobenzene	49	269	75	100 h	Liquid	^a^	^a^	[16]
PzAzo ether 8ene (B8)	^a^	329	^a^	90d	Liquid ↔ Solid	5	0.95	[79]
T-Azo	^a^	171	^a^	^a^	Liquid ↔ Solid	10	0.74	[86]
F-Azo	^a^	198	^a^	^a^	Liquid ↔ Solid	10	0.72	
1	77	134	37	^a^	Liquid ↔ Solid	^a^	0.60	[59]
2	88	100	28	^a^	Liquid ↔ Solid	^a^	0.74	
3	87	108	30	^a^	Liquid ↔ Solid	^a^	0.80	
MO-Azo@DMBI 50:1	^a^	205	57	^a^	Liquid ↔ Solid	15	0.99	[90]

^a^No data was found in the references

**Fig. 7:**
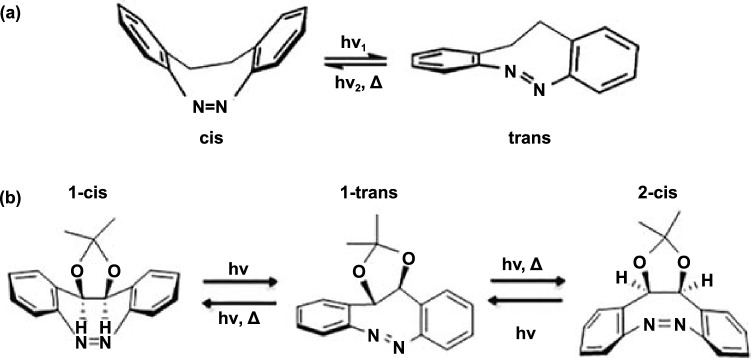
Scheme **a** [91] and scheme **b** [93]

It is worth noting that bridging azobenzene derivatives’ unique properties, such as higher quantum yield and photoisomerization ratio, can also enhance the Azo-STFs’ performance, which have reignited the interest of the scientific community in recent years. Siewertsen [91] reported the photoswitching behavior of 5,6-dihydrodibenzo[c,g][1,2]diazocine (Fig. 7 Scheme a) in 2012. He showed that *cis* can be switched to *trans* with an efficiency of > 90% using blue light at *λ* ≈ 370–400 nm, and *trans* can be switched back to *cis* with nearly 100% efficiency using green light with *λ* ≈ 480–550 nm. A > 90% photoconversion yield cannot be achieved in the case of azobenzene. However, the *n*–π* absorption of the *trans* isomer is stronger than that of the *cis* isomer. The 4.5 h thermal lifetime of *trans* at room temperature is not a drawback, especially for applications involving fast repeated forward and backward switching cycles or at lower temperatures. Four years later, Gao et al. [92] have run trajectory-based nonadiabatic on-the-fly molecular dynamics simulations on the bridged-azobenzene photoisomerization for both *cis*-to-*trans* (400 sampling trajectories) and *trans*-to-*cis* (550 sampling trajectories) at the SA2-CASSCF(6,5)/6-31G quantum chemistry level. They found that the enhanced photoresponsibility of *trans*-b-Ab, such as the higher quantum yields and shorter excited state lifetimes than in the parent azobenzene, originates from the steeper PES in the S1 state, shorter isomerization pathway, and favorable nonplanar initial structure. Poyer et al. [93] used liquid chromatography coupled with UV detection combined with ion mobility–mass spectrometry to study the thermal back relaxation kinetics of a bridged azobenzene derivative (Fig. 7 Scheme b). The multi-isomerization process endows bridging azobenzene with a multilayered energy storage mechanism, enabling it to release heat continuously and controllably from either 1-*cis* or 2-*cis* to 1-*trans*. The properties bridging azobenzene derivatives exhibit deserve further research.

### Azobenzene with Nanocarbon Template

The enthalpy of Azo molecules can be adjusted through intermolecular forces (number and bond strength), intramolecular electronic interactions, and steric hindrance. The resulting impact mainly depends on the combined effect of the functional group (electronic effect, number, and position) of the Azo molecule, molecular orientation, spatial configuration, and distance between molecules. Although the modification of azobenzene with derivatives can result in an increase in the enthalpy of photoisomerization, owing to the features of azobenzene molecule, modification using this method does not result in the ideal state. It has been found that high Δ*H* and long *t*_1/2_ are difficult to attain simultaneously in a single Azo molecule only by changing the substituents. Therefore, at the moment, when no breakthroughs have been reported in the research on increasing the enthalpy of photoisomerization of azobenzene derivatives, researchers are focusing on the enthalpy change using intermolecular interaction force. The disordered arrangement of single-molecule azobenzene makes the enthalpy of intermolecular interaction less effective. In fact, the modification of azobenzene molecules can be further expanded on. Compared with random disordered azobenzene derivative single molecules, the templated structure of Azo molecules is more conducive to improving intermolecular interactions and sterically hindered close-packed ordered structures, which can significantly increase the Δ*H* and *t*_1/2_ of azobenzene. The template method not only improves the energy storage capacity of Azo-STFs but also endows azobenzene with better physical properties. Compared with azobenzene derivatives, membrane energy storage materials prepared based on azobenzene templates have better physical properties and therefore potentially have broader application scope.

Carbon nanomaterials have excellent electrical/thermal conductivity and can be combined with other non-conductive materials into conductive fabrics or films, which are widely used in machinery, electrics, and chemistry. In fact, graphene, a two-dimensional carbon material, can show ultra-high thermal conductivity (TC) of 4800–5300 W (m K)^−1^, far exceeding that of graphite (currently up to 2000 W (m K)^−1^) [94–96]. At present, researchers often use two-dimensional carbon materials in composites [97–101].

Regarding azobenzene nanocarbon template materials, currently three carbon nanomaterials are mainly researched: graphene, reduced graphene oxide, and carbon nanotubes. One-dimensional or two-dimensional nanocarbon templates play a supporting role and are considered an excellent platform for assembling tightly arranged ordered Azo molecules. The interaction between the ordered azobenzene molecules reduces the energy of the *trans* isomer while stabilizing the *cis* isomer, which leads to a significant increase in the Δ*H* and *t*_1/2_ of the molecule [66]. Intermolecular interactions and steric hindrance are controlled by steric configuration and functional groups. Therefore, high-density grafting of azobenzene molecules on graphene is considered a feasible strategy for improving the storage performance of STFs [68]. Although the molecular aggregation of Azo molecules can hinder isomerization and cause long-term photon absorption, the charge transfer between Azo molecules and graphene can also catalyze isomerization. Hence, the molecular aggregation of Azo molecules has little effect on the power performance of STFs [102–107].

**Fig. 8:**
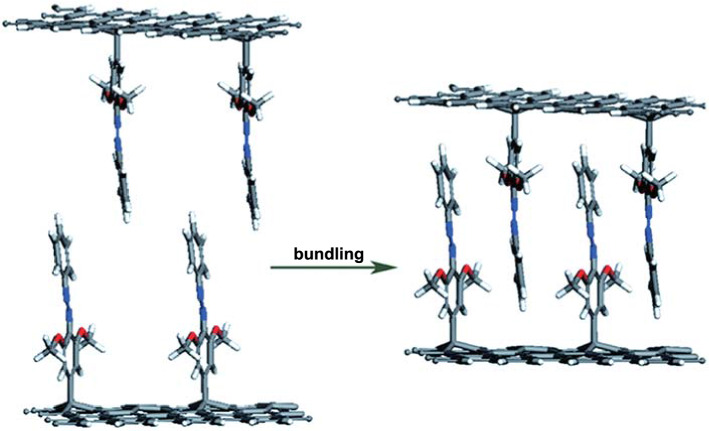
Illustration of the mechanism of inter-planar bundling for high functionalization density Azo–RGO hybrids, which show no packing interaction in the solution (left) because of a large interlayer spacing but show packing interaction in the solid state forms because of the bundling with a low interlayer space (right) [112]

Based on the DFT calculations, it was found that molecular interactions such as hydrogen bonding and proximity-induced interactions can significantly increase the Δ*H* and *t*_1/2_ of each Azo molecule [101, 108]. With proper intermolecular separation, the interaction between the *π* electrons of benzene rings adjacent to *trans*-Azo molecules results in a net increase of 0.2 eV in Δ*H* per molecule. However, these calculations are mainly based on nanocarbon templates with high functionalization density. This type of template may be used to extend solar thermal storage and attain high ED by combining the increased number of intermolecular interactions per unit volume and the increased weight per unit storage capacity. Compared with the theoretical model (1/4–1/8), the lower storage capacities of these nanomaterials are mainly attributed to the low Δ*H* and low functional density of the single Azo molecule, which is 1/40–1/80 (each Azo molecule consists of 40–80 carbon atomic supports) [109–111]. To solve this, Feng et al. [112] innovatively designed a nano-template for high functional density Azo-STFs, which is composed of Azo molecules with methoxy and/or carboxyl groups covalently attached to the surface of graphene nanosheets (Fig. 8). The authors of this paper proved that this solar storage nano-template (Azo-RGO) uses intermolecular hydrogen bonding and proximity-induced interaction to achieve high storage capacity (112 Wh kg^−1^), long-term storage life (*t*_1/2_ of 33 d), high functional density, and solid intercrystalline binding with stacking interaction. In addition, this STF exhibits strong cycling performance in 50 cycles and can provide at least 4.5 years of long-term use. Our group then used DFT to research the Δ*H* of different groups of Azo/graphene models and found that the electron-withdrawing groups (–NO_2_, –SO_3_H, and –COOH) at the ortho position of active benzene (AB) can increase Δ*H* by stabilizing the *trans* isomer at low energy. Feng et al. attributed this effect to the resonance effect [113].

It is worth noting that high-density cross-linking of hydrogen bonds usually introduces a considerable thermal barrier to isomerization, resulting in a low degree of reversible isomerization (< 64%) [107, 114]. In addition, owing to the low selectivity, hydrogen bonds tend to reform before recovery. Therefore, the bonding enthalpy is released spontaneously during the transition, and the lack of control of formation/dissociation limits further improvement in heat storage. In contrast, coordination bonds (dynamic metal–ligand interactions) have shown great potential in expanding the energy gap because of their relatively high binding energy and controllable formation/dissociation [115]. This optically triggered reversible formation/dissociation is necessary for heat storage and release caused by isomerization. In addition, relatively suitable bond strength is not only conducive to reversible interaction with organic ligands but also conducive to structural transformation owing to relatively low steric hindrance [116]. A large number of coordination bonds can provide high bond enthalpy through coordination and dissociation during the isomerization. This implies that further research on coordination bonds may provide important insights for solving potential problems in the field of closed-loop utilization of photothermal energy.

**Fig. 9:**
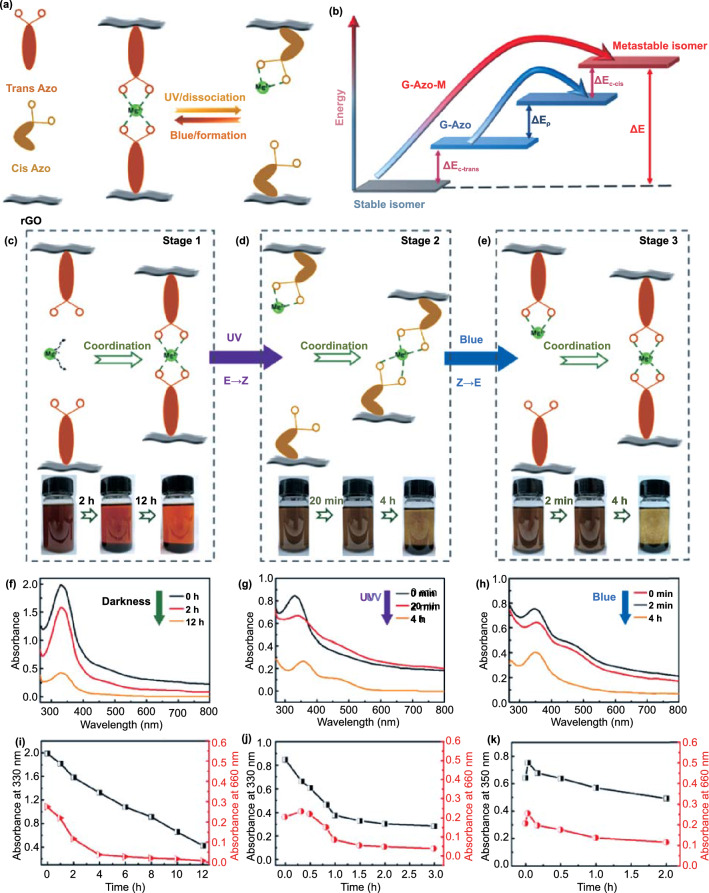
**a** Schematic of reversible photoisomerization of G-Azo-Mg film in the solid state with formation and dissociation of coordination bonds. **b** Schematic energy diagram of G-Azo tuned using coordination bonds. Reversible formation and dissociation of coordination bonds in liquid state. Schematic of the reversible formation and dissociation of G-Azo-Mg for crosslinked self-assembly in DMF solution in **c** stage 1, **d** stage 2, and **e** stage 3. The corresponding photographs of the solution at different time points indicate the dissolution (disassembly) and deposition (assembly). **f**–**h** Time-evolved UV–Vis absorption spectra of the solution in stage 1, 2, and 3, respectively, under alternate irradiation of UV and visible lights. **i**–**k** Absorption intensity at 330 and 660 nm with elapsed time in stage 1, 2, and 3, respectively [117]

Based on this, Feng et al. [117] introduced a new type of reversible dynamic bonds into a nano-graphene-templated azobenzene (G-Azo) hybrid. G-Azo spontaneously formed a loose layered self-assembly (denoted as G-Azo-Mg), cross-linked by Mg-O coordination bond or electrostatic bond formed between Mg metal ion and sulfonate, demonstrating the grafting density as high as 1:21 (Fig. 9). Under alternating UV and blue light irradiation, the optically triggered solid-state formation and dissociation of the coordination bond cause the expansion of the molecular energy gap through a reduction in the energy level of the *trans* isomer, and the accelerated reversal of the unstable *cis* isomer can be achieved by increasing the energy level. Compared with other G-Azo-M hybrids, owing to a large number of Mg–O bonds and relatively high Δ*E* (4.33 eV), the solid-state G-Azo-Mg module has a wider operating temperature range (0.0–25.0 °C). G-Azo-Mg-3 demonstrated the maximum ED (up to 720.1 kJ kg^−1^), and the film made of it can quickly release latent heat, demonstrating a high PD of 2871.1 W kg^−1^, and the heat output rate can be adjusted by blue light irradiation at different temperatures. The studies discussed above have provided a sufficient experimental basis for the development of Azo/graphene high-density STFs by controlling molecular interactions.

**Fig. 10:**
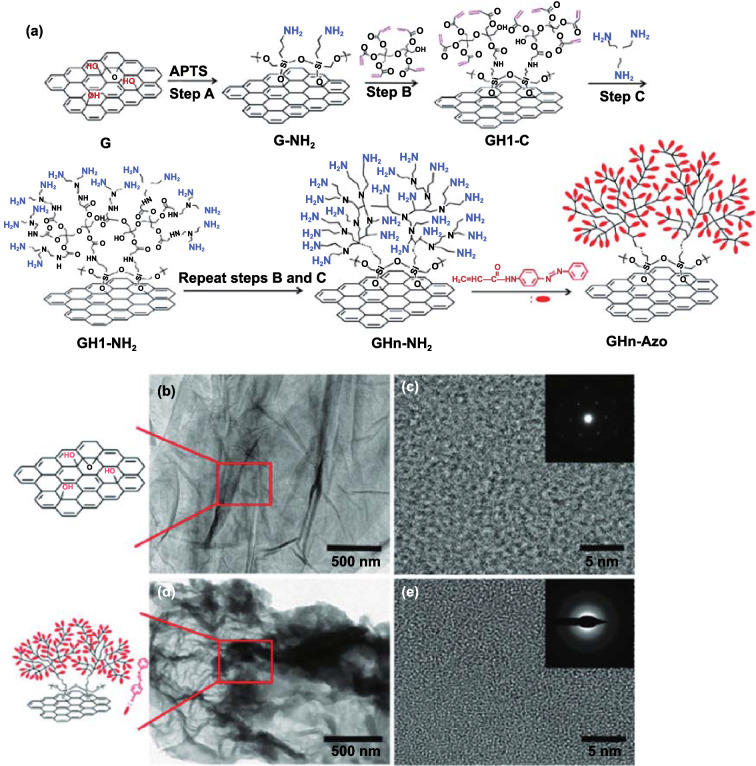
**a** Schematic of azobenzene–graphene STFs with hyperbranched structures. G-NH2: graphene hybrids terminated with amine groups via coupling treatment; GH1-C: graphene hybrids terminated with acrylate groups via Michael addition reaction; GHn-NH_2_: graphene hybrids terminated with multitudinous amine groups via layer-by-layer dendritic amplification; n represents the factor of dendritic amplification. GHn-Azo: azobenzene–graphene hybrids with different amplification layers. TEM images and chemical structures of graphene and GH3-Azo hybrids. **b** Low-resolution and **c** high-resolution images of graphene. **d** Low-resolution and **e** high-resolution images of GH3-Azo hybrids. Inset: FTT patterns [118]

It is worth noting that the storage performance of Azo-STF can also be improved through the increase in the number of layers. It was reported that Xu et al. [118] grafted a large number of primary amine groups onto the surface of graphene through hyperbranched layer-by-layer deposition and then let them react with acryloyloxyazobenzene molecular coupling to graft a large number of azobenzene molecules on graphene using surface amplification technology. Among them, the ED of the three-layer hyperbranched azobenzene-graphene hybrid reached 104 Wh kg^−1^ (374 J g^−1^), which is three times that of the original azobenzene. They attributed this phenomenon to increased steric hindrance and molecular interaction between adjacent azobenzene molecules. In addition, the storage half-life of the three-layer azobenzene–graphene hybrid prepared by Xu et al. reached 80 h, which was 30 times longer than that of the original azobenzene (2.5 h) (Fig. 10).

**Fig. 11:**
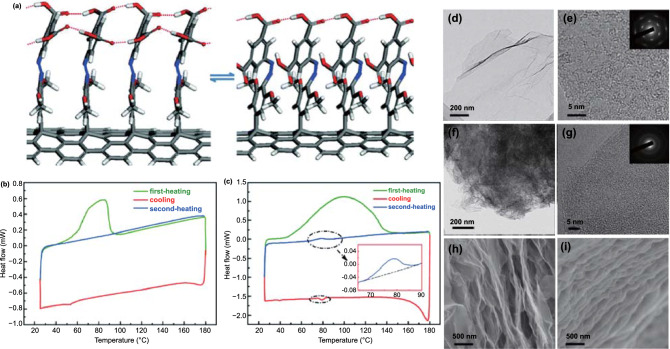
**a** Stable models of *trans* and *cis* isomers of Azo–RGO-1 obtained using DFT calculations. DSC traces of **b** Azo and **c** Azo–RGO-1 with first-heating (green), cooling (red) and second-heating (blue) periods. TEM images of **d** RGO with **e** a high resolution, **f** Azo–RGO-1 with **g** a high resolution and SEM images of **h** RGO and **i** Azo–RGO-1 [100]. (Color figure online)

To make better use of intermolecular forces, researchers turn their attention to GO. As a grafted functional group of graphene, graphene oxide (GO) also has a two-dimensional sp^2^ hybrid network, in which *π* electrons are delocalized on the ring [119]. The difference is that GO, as a functionalized graphene, contains carboxyl, hydroxyl, and carbonyl groups at the edge and epoxy and hydroxyl groups at the base. Given that the edges and surfaces of GO are coated with –OH groups, it is reasonable to use GO as a coupling agent to obtain Azo-GO hybrids. Based on this consideration, Pang et al. [120] developed a method to covalently link the azobenzene moiety to GO and created a chromophore/graphene oxide (Azo-GO) hybrid. This hybrid material has a high ED of nearly 240 Wh kg^−1^, which may be attributed to the high grafting density of the Azo part and low quality of the new structure. It is worth mentioning that the numerous functional groups can not only increase the energy storage capacity of the GO template but also reduce the cycling stability of the template. To balance stability and energy storage performance, researchers have gradually turned their attention to reduced graphene oxide (RGO). While the RGO template retains the stability of the graphene template, it also possesses part of the functional groups of the GO template. Feng et al. [100] prepared an STF based on molecular nano-templates with high grafting density. The molecular nano-templates consist of graphene and azobenzene, which is bound to the surface of the graphene covalently (Azo-RGO-1), as shown in Fig. 11. The grafting density of Azo-RGO-1 hybrid is as high as 1/17. The intermolecular hydrogen bonding and stacking interactions in the template significantly improved the ED (138 Wh kg^−1^); moreover, the material possessed a longer storage life (*t*_1/2_ = 52 d) and excellent cycling stability under 520 nm visible light (50 cycles).

**Fig. 12:**
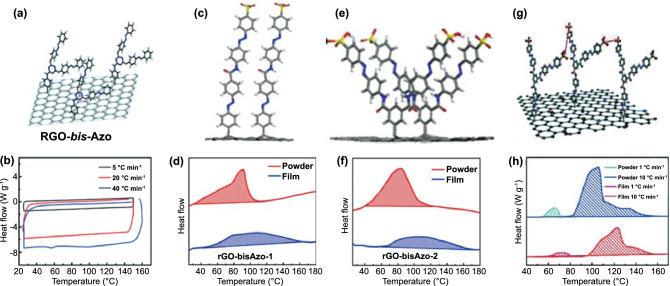
**a**, **b** Chemical structure and DSC traces at the *cis*-rich photostationary states after the irradiation by UV light with the first heating at different rates of RGO-bis-Azo [123]. **c–f** Chemical structures and DSC traces in the first heating stage of RGO-bisAzo-1 and RGO-bisAzo-2 [107]. **g**, **h** Chemical structure and DSC traces of the tri-Azo1:68/RGO powder and film at heating rates of 1 and 10 °C min.^−1^, respectively [114]

Strong steric hindrance usually hinders the uniform and high-density lattice between Azo molecules and carbon templates. To solve this problem, Kucharski further replaced the monoazo unit with the polyazo unit [66]. In fact, as photoisomerization is simultaneously affected by intramolecular and intermolecular interactions, the polyazo moiety is considered one of the ideal chromophores for solar thermal storage. Theoretically, bis/tris-Azo molecules show higher storage density and longer storage time. Friedrich et al. [121, 122] reported that owing to molecular interactions, the intramolecular interactions of bis/tris-Azo lead to drastic changes in the energy and geometric structure of the isomers. In addition, a greater number of Azo units in a molecule result in a more thermally stable *cis* isomer. Feng et al. [123] reported on 2-chloro-4,6-bis(4-(phenyldiazenyl) phenoxy)-1,3,5-triazine (bis-azobenzene chromophores) closely packed with RGO (RGO-bis-Azo) (Fig. 12a, b). The spatial configuration and energy of the *trans* and *cis* isomers of bis-Azo are affected by the intramolecular and intermolecular steric hindrance between two adjacent Azo units on the same and adjacent nanosheets. This resulted in the RGO-bis-Azo hybrid possessing a combination of high ED of 80 Wh kg^−1^, maximum power density of 2230 W kg^−1^, an adjustable heat release time from 2 min to 5520 h (*t*_1/2_ of 1320 h) and good cycle performance (50 cycles) owing to the high grafting density and bundling effect. Based on the above study, Feng et al. [107] further prepared two thin films assembled from close-packed bis-Azo grafted onto RGO to obtain a photochromic material with a perfect combination of high energy and stability, as well as controllable solid-state heat release trigger (Fig. 12c–f). They studied the closed loops of light collection, storage, and heat release of these two films. One of the assembled RGO-bis-Azo templates exhibited a high ED of 131 Wh kg^−1^ and a long half-life of 37 d and still had good cycle performance after 50 cycles. In addition, at an ambient temperature difference greater than 100 °C, the RGO-bis-Azo-2 film released and accumulated heat, which allowed it to attain a maximum temperature difference (Δ*T*) of 15 °C and Δ*T* above 10 °C for more than 30 min. The controlled heat release in solid-state components paves the way for the development of high-efficiency and high-energy photothermal fuels (PTFs) for a variety of applications.

Push–pull groups have been reported to increase the difference in dipole moments between *trans* isomerization and *cis* isomerization azobenzene. Therefore, the multi-branched Azo assembly facilitates the formation of intramolecular/intermolecular interactions and increases the energy difference (Δ*H*) between the *trans* and *cis* isomers. In addition, the component can also control the *trans* → *cis* isomerization of different Azo units (free or limited) by changing the molecular structure. As a result, Feng et al. [114] produced a templated component (tri-Azo1:68/RGO) based on tris-azobenzene, which has a maximum ED of 150.3 Wh kg^−1^, long half-life (1250 h), and high power density of 3036.9 W kg^−1^ (Fig. 12g, h). The film made of tri-Azo1:68/RGO can release 23.6–69.7% of the stored heat, which is accompanied by a temperature increase of 2–7 °C, which successfully optimizes the solid-state high-speed heat output, enabling the use of photothermal energy for the closed-loop utilization of thermochromic displays. Subsequently, they applied the heat output to the thermochromic display by effectively causing the color of the pattern to change at a temperature lower than the CT. The high resolution and wide applicability of thermochromic displays indicated that the film can uniformly release heat at different rates. Even when stored at a low temperature of 0 °C for 5 d, the utilization efficiency of tri-Azo1:68/RGO membrane still reached 10.2%. This closed-loop efficient utilization can be used as an excellent source of thermal power for thermal management, which provides insights for the development of STF films. However, although great progress has been made in improving ED and adjusting storage life, there is still a big gap between the performance of the photosensitive chemical heat storage materials composed of pristine azobenzene and the requirements of actual applications. To optimize the low-temperature exothermic capabilities of azobenzene, Yang et al. [124] covalently linked fluorinated azobenzene (AzoF) to RGO and prepared a photosensitive chemical heat storage material (AzoF-RGO). The test results showed that the AzoF-RGO composite can release all of the stored energy within 2 min at 45 °C, which is a high rate of heat release with short-term rapid concentrated heating at low temperatures. This study provides a solid foundation for the commercialization of azobenzene solar fuel using graphene as a template in the future.

**Fig. 13:**
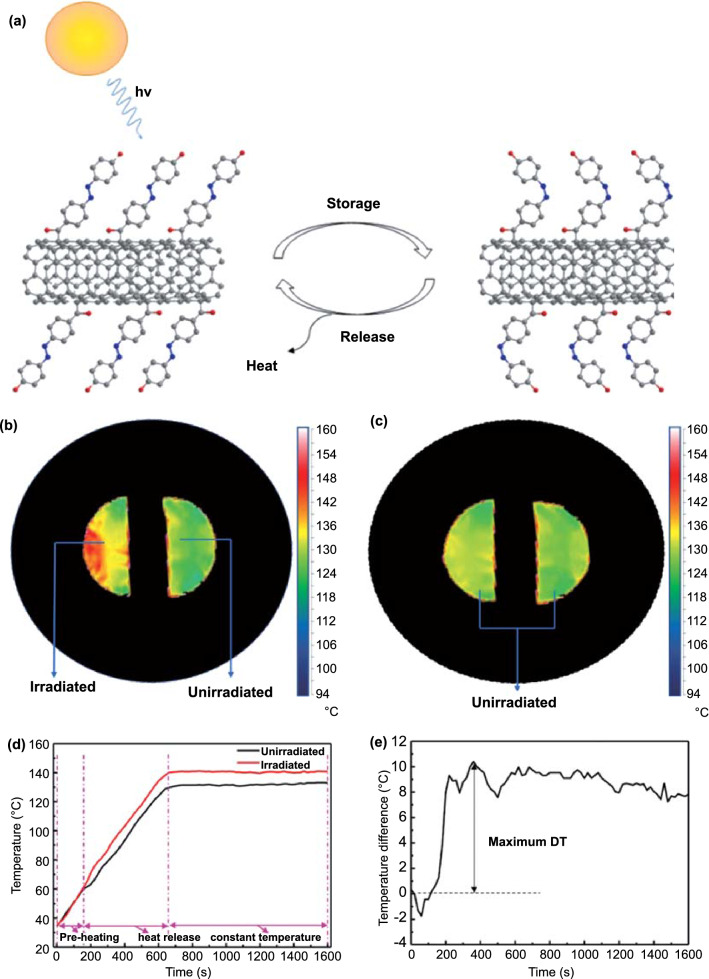
**a** Schematic of the Azo-SWCNTs STFs based on the photoinduced isomerization. **b** Azo-SWCNTs film with and without irradiation. **c** Both Azo-SWCNTs films absorbed no UV light. **d** Average temperature on the surface of Azo-SWCNTs film. **e** Average DT between Azo-SWCNTs film with and without irradiation [125]

Although the Azo-graphene (Azo-G) hybrid has a large enthalpy of isomerization, it requires long-term UV irradiation (at least a few hours) to store energy, resulting in low absorption capacity and low conversion rate. In this light, the attention of the scientific community began to shift from graphene templates to carbon nanotube templates (Azo-CNT). Grossman et al. [66] prepared Azo single-walled carbon nanotubes (Azo-SWCNTs) without push–pull electronic structure and hydrogen bond regulation. The results demonstrated that Azo-SWCNTs has higher energy stored per Azo molecule by more than 30% (56 Wh kg^−1^) and a good half-life (*t*_1/2_ ≈ 33 h), which enables its application as a short-term daily STF. However, the current ED of Azo-CNTs STFs limits future applications. In addition, Azo-CNTs STFs usually exist in powder form and need to be diluted with organic solvents to store energy, which also limits their practical application. For actual large-scale STF applications or thermochromic display applications, solid films are more useful than powders [114]. Therefore, Jiang et al. [125] prepared a solid-state STF molecule composed of single-walled carbon nanotubes and azobenzene (Azo-SWCNT) with a push–pull electronic structure through a direct Friedel–Crafts acylation reaction (Fig. 13). The material showed the highest weight ED (80.7 Wh kg^−1^) and relatively higher power density (292 W kg^−1^) in the STF system and had a longer half-life (16 h) and excellent photochemical cycling stability. This study concluded that solid-state STF thin films made of such nanostructured hybrid materials have great potential for efficient and reversible solar thermal storage and are worthy of further research. The performance comparison of the azobenzenes with different nanocarbon templates has been illustrated in Table 2.

**Table 2:** Parameters related to nanocarbon-templated Azo-STFs

Nanocarbon-templated Azo-STFs	Energy density			*t*_1/2_	State	Grafting density	Cycles	Charging degree	References
	kJ mol^−1^	J g^−1^	Wh kg^−1^						
Azo-RGO	192	403	112	33 d	Solid^b^	1/16	50	^a^	[112]
G-Azo-Mg-3	^a^	720	^a^	96 h	Solid^b^	1/21	10	0.71	[117]
GH3-Azo	94	374	104	80 h	Solid	1/30	20	0.26	[118]
Azo-GO	174	^a^	240	10 h	Liquid	1/16	^a^	0.60	[120]
Azo-RGO-1	265	497	138	52 d	Liquid	1/17	50	^a^	[100]
RGO-bis-Azo	318	288	80	1320 h	Solid^b^	1/50	50	0.65	[123]
RGO-bis-Azo-1	412	346	96	1120 h	Solid	1/59	50	0.84	[107]
RGO-bis-Azo-2	572	472	131	900 h	Solid	1/46	50	0.73	
tri-Azo1:68/RGO	943	^a^	150	1250 h	Solid	1/68	100	0.88	[114]
Azo-SWCNT	^a^	^a^	81	16 h	Solid	1/31	30	0.64	[125]

^a^No data was found in the references

^b^Suspended in solvent

In addition, Telgerafchi et al. [126] reported that templated azobenzene molecules on the sidewalls of functionalized multi-walled carbon nanotubes (MWCNT) can also increase the energy stored by each azobenzene molecule, which indicates that in addition to single-walled carbon nanotubes, multi-walled carbon nanotubes can also be applied in hybrid STFs.

Interestingly, a promising type of materials which can be used for carbon nanomaterials—Metal–organic frameworks (MOFs) was found after summarizing the literature. MOFs bring almost unlimited possibilities owing to their "designable" potential [127]. The overall structure, porosity, and surface functional groups of MOFs can be designed, synthesized, and adjusted to meet specific application needs. MOFs have been deeply explored for potential applications in the storage and separation of molecules [128, 129]. The azobenzene functional group can change its conformation when treated with light or heat. This isomerization involves changes in the distance between the para-carbon atoms in the azobenzene molecule from 9 Å in the *trans* isomer to 5.5 Å in the *cis* isomer, which can be introduced into the organic linker of MOFs to perform photomechanical tasks at the molecular level [130–132].

**Fig. 14:**
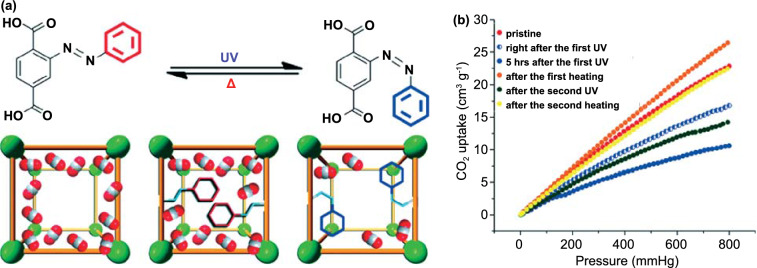
**a** (Top) *Trans*-to-*cis* isomerization of the ligand of PCN-123 induced by UV irradiation and the *cis*-to-*trans* isomerization induced by heat treatment. (Bottom) Schematic illustration showing the suggested CO_2_ uptake in MOF-5, PCN-123 *trans*, and PCN-123 *cis*. **b** CO_2_ adsorption isotherms (at 295 K) of PCN-123 showing reversible conformational change [133]

Zhou [133] used a ligand, 2-(phenyldiazenyl)terephthalate, to synthesize a photoswitchable MOF and named it PCN-123 (PCN represents porous coordination networks, Fig. 14). The crystals of PCN-123 exhibit pseudo-high symmetry. Finally, Zhou found that PCN-123 can adsorb different amounts of CO_2_ after UV or heat treatment, which also proved that Azo-MOFs can potentially be used in photoswitches. Recently, the optical absorption spectra of the *cis* and *trans* configurations of PCN-123 were calculated using the Bethe–Salpeter equation formalism and GW approximation using periodic and nonperiodic models. Calculations by Aseem place the largest selective absorption by *cis* isomer at around 3.2 eV and by *trans* isomer at nearly 4.2 eV [134]. Aseem found that the most important difference between the molecular and periodic models is the ratio between the oscillator strength of *cis* and *trans* isomers for the S1, which is significantly larger in the periodic MOFs than in any fragment model, including the free azobenzene molecule, as discussed above, which can be attributed to the limitation of nonperiodic models to provide a quantitative description of the electronic structure of the periodic system at the DFT level.

**Fig. 15:**
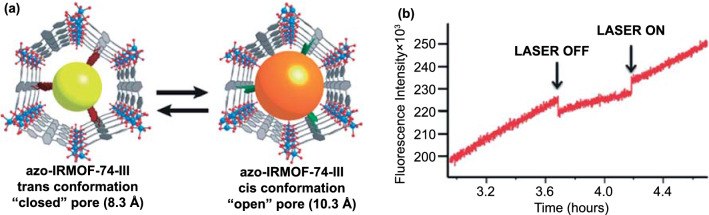
**a** View of the idealized azo-IRMOF-74-III down the c-axis displays one-dimensional pores and azobenzene functional groups projecting into the pores. The yellow and orange balls represent pore aperture in idealized Azo-IRMOF-74-III, when the azobenzene functional groups are in *trans* and *cis* conformation, respectively. **b** An on/off trial, where the excitation beam was temporarily removed mid-release [135]

Azo-MOFs can adsorb not only gases but dyes, alkanes, and other molecules. The control of the guest release from the pores in response to an external stimulus has been demonstrated for azobenzene functionalized isoreticular metal–organic framework (Azo-IRMOF-74-III) [Mg_2_(C_26_H_16_O_6_N_2_)] by Brown et al. [135] Azo-IRMOF-74-III is an isoreticular expansion of MOF-74 with an etb topology and a 1-D hexagonal pore structure (Fig. 15). When propidium iodide dye was loaded into the MOF, spectroscopic studies showed that no release of the luminescent dye was observed under ambient conditions. Upon irradiation of the MOF at 408 nm, however, the rapid wagging motion inherent to the repetitive isomerization of the azobenzene functionality triggered the release of the dye from the pores. The light-induced release of cargo can be modulated between on and off states using light of an appropriate wavelength.

Photoswitching has different effects on the adsorption of different guest molecules. Wang [136] used thin surface-mounted MOF films to investigate its impact on the adsorption properties of various guest molecules in nanoporous, azobenzene-containing MOFs (Cu_2_(BDC)_2_(AzoBipyB)). Wang found that the impact of the photoswitching of azobenzene in the MOF structure on the adsorption properties greatly depends on the guest molecules; the uptake of polar molecules was significantly increased by the switching of azobenzene side groups to the *cis* state. This study can help adjust smart materials for specific advanced applications.

The poor cycle performance of Azo energy storage system may be caused by its frequent isomerization during cycling. Template method, one of the most actively researched directions, consists of “inserting” the Azo unit into the carbon nano-template by grafting. However, frequent isomerization may cause irreversible damage to the chemical bonds or the structure of the Azo unit, and there are no effective means of replacing it on the carbon nano-template. Azo-MOF materials provide important insights on combining azobenzene derivatives and the template method. Insertion of azobenzene into Azo-MOFs template as a guest can enable full use of coordination bonds and other intermolecular forces while realizing the replaceability of azobenzene molecules. This new strategy is very interesting and can potentially be realized.

In addition, azo energy storage systems require good film-formation properties to ensure full utilization of solar energy during the charging process. Since MOFs are usually grown as single crystals or polycrystalline powders, a thin film of optical-switch-integrated MOF should be prepared, which has become a hotspot in recent years. In 2017, Meller [137] presented the first Azo-MOF, the key properties of which may be reversibly modulated using irradiation with visible light only. The fluorinated azobenzene side groups in the MOF structure can be reversibly switched between the *trans* and *cis* states using green and violet light (not requiring the use of UV light). Interestingly, Azo-MOFs have certain film-forming properties. Wang [138] used a combination of surface-mounted MOFs (SURMOFs) with a quartz crystal microbalance to prepare five different photoswitchable azobenzene-containing pillared-layer MOF structures. This study also revealed a trend that a large switching effect is reached for a high density of azobenzene moieties per MOF unit cell. Khayyami et al. [77] demonstrated in situ fabrication of crystalline thin films from various azobenzene-based metalorganic structures using atomic/molecular layer deposition; these are highly intriguing materials as azobenzene is one of the prototype organic molecules that undergo photoisomerization. In 2020, Rice [139] published the first review that solely emphasizes basic photophysical research and its dependence on the type of MOF or photoactive part. This review showed several key challenges in the development of Azo-MOFs, which are considered a new type of smart material.

The photoswitching mechanism of Azo-MOFs show a possibility which can be introduced to Azo-STFs with carbon nano-templates, allowing azobenzene derivatives or catalysts to disperse between the carbon nano-templates layers controllably. This may endow Azo-STFs with flexible replaceability while realizing multi-dimensional heat storage and provide a possibility for composite and controllable energy release by combining template-azobenzene, azobenzene derivatives and catalyst, which can also be used for the realization of Azo-STFs.

## Controllable Azobenzene Energy Release

Although energy storage capacity is one of the most important criteria for Azo-STFs, it is not the only important factor for the development of Azo-STFs. The operating mechanism of Azo-STFs can be roughly divided into two parts: "charging" and "discharging." During charging, Azo-STFs absorb photons in the UV region of the electromagnetic spectrum and transform from low-energy, stable *trans* isomers to high-energy, metastable *cis* isomers. This metastable “charged” state can be maintained for a few hours to a few years. Under external stimulation, the metastable *cis* isomer can overcome the energy barrier, complete the discharge process, and return to the thermodynamically stable *trans* isomer [140]. To deal with unexpected circumstances that may occur during daily use, controllable Azo-STFs are vitally important.

As mentioned in Sect. 2, the isomerization of azobenzene molecules is easily affected by the environment. Factors that can affect the isomerization of the Azo unit include but are not limited to light, heat, catalysts, solvents, electric field, magnetic field, stretching, etc. The great development prospect is one of the main reasons for the attention of Azo-STFs. It is necessary to explore the exothermicity of azobenzene in multiple scenarios. The isomerization process of azobenzene is known to be exothermic/endothermic. Therefore, exploring the isomerization process of azobenzene under different conditions is necessary for further exploration of Azo-STFs in the future. In recent years, multiple related studies have been published, but their content has rarely been associated with energy storage. Azobenzene materials are the most commercially promising photothermal energy storage PCMs, and while their energy storage performance is gradually improved, their controllable energy release deserves more attention.

### Catalyst

**Fig. 16:**
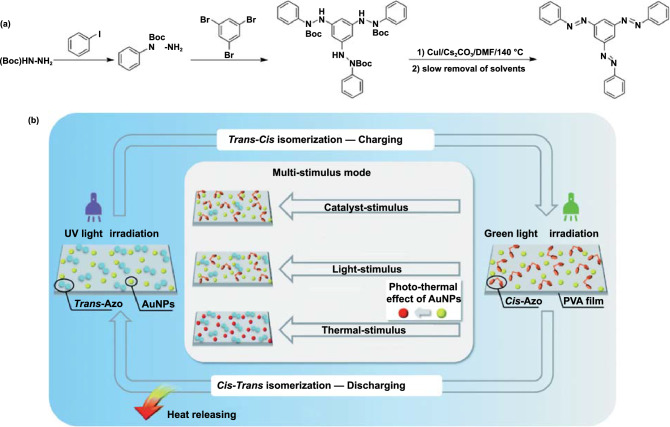
**a** Route used to synthesize tri-azobenzene. **b** Schematic of the charge–discharge procedure of PVA/Azo@AuNP STF [140]

The discharge of Azo-STFs can be effectively controlled using catalysts. Under normal circumstances, the *cis*-to-*trans* isomerization of azobenzene under thermal catalysis (reverse isomerization) is completed in a period from several hours to a few days. However, the presence of gold nanoparticles can accelerate the deisomerization of azobenzene to a few minutes [141–144]. The activation energy required for the *cis*-to-*trans* thermal relaxation of the azobenzene derivative on the Au(111) surface can be reduced by 75% compared to that in the solution [145–148]. Santer et al. [149] found that in the presence of gold nanoparticles, the thermal life of the *cis* isomer containing azobenzene molecules can be reduced by up to 3 orders of magnitude compared with the life in the solution without nanoparticles. In addition, Feng et al. [140] found that gold nanoparticles can also enhance the energy storage capacity of azobenzene solid STF films templated with polymers (Fig. 16). The heat storage and release behavior of the PVA polymer-templated, gold nanoparticle (AuNP) doped, 1,3,5-tris(arylazo)benzene (tri-Azo)-based STFs (PVA/Azo@AuNP STFs) presented by Feng et.al can be completely controlled using light alone at room temperature. Light with a wavelength of 365 nm is effective for charging the PVA/Azo@AuNP STFs, while 520-nm light induces the release of energy from the samples. Temperature increases of up to 8.8 °C were observed during the discharge of PVA/Azo@AuNP STFs.

**Fig. 17:**
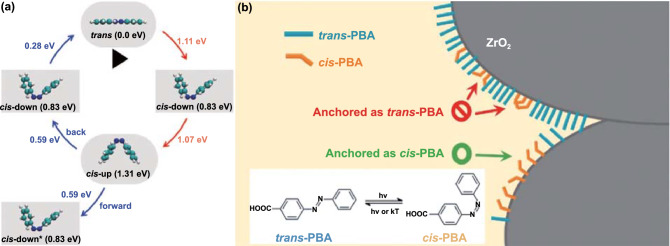
**a** Catalytic mechanism of AuNPs [150]. **b** Catalytic mechanism of ZrO_2_ nanoparticle films [151]

In 2020, Xie et al. [150] carried out first-principles simulations to map the complete isomerization path for the AB molecule adsorbed on the Au(111) surface (Fig. 17a). Their results suggest a new metastable configuration as the in situ *cis*-AB, which can well reproduce all the experimental measurements in the STM observations. This means that Xie et al. identified a new pathway for the *trans*–*cis* isomerization on the Au(111) surface.

Angelini et al. [152] investigated the effect of these uncoated negatively charged AgNPs on the thermal *cis*–*trans* isomerization of azobenzene and found that 4‑methoxyazobenzene (MeO-AB), resonance-activated derivative of azobenzene, added to the AgNPs aqueous solution in *trans*-state was converted into the *cis*-form by irradiation immediately or 120 h after the preparation of the samples. Interestingly, the use of silver nanoparticles as a catalyst or reaction medium seems to be limited because in the absence of any stabilizers, silver nanoparticles rapidly oxidize and aggregate. The study by Angelini et al. provides a possibility for solving such problems.

In addition to nanoparticles, metal oxide nanoparticle films are also among the preferred supports for Azo-STF energy conversion devices. Achey et al. [151] found that the interface attachment of azobenzene derivatives with ZrO_2_ nanoparticle films can double the rate of azobenzene thermal isomerization and photoisomerization and reduce the degree of transformation by a factor of 3, compared with those in fluid solutions (Fig. 17b). More interestingly, the magnitude of these changes can also be controlled by selectively anchoring the *cis* or *trans* isomer of azobenzene to the ZrO_2_ membrane.

Interestingly, the isomerization of azobenzene may also be affected by metal ions and acid radical ions, including sodium, copper, bromide, chloride, and other ions [153]. Generally speaking, the catalytic ability of cuprous ions for the isomerization of azobenzene is not outstanding; it is much lower than that of gold nanoparticles. However, it has been discovered that divalent copper ions can have a significantly greater catalytic effect than cuprous ions. Recently, Hossain [154] reported two new macrocyclic azobenzene materials that showed, in addition to their normal optical switching behavior, an unusual instantaneous transition from the Z type to the E isomer in the dark at 273 K and in the presence of Cu^2+^ ions. Cu^2+^ complex can maintain Z-isomer radiation under constant UV light. However, once the exposure to UV light ceases, it reverses to type E. The same phenomenon was observed with Ag^+^ ions but at a slightly lower speed.

In short, many catalysts that can affect isomerization have already been identified for different azobenzene derivatives. Most related articles chose to disperse gold nanoparticles as catalysts into the interior of Azo-STFs to achieve the controlled release of energy. In the above, we analyzed a variety of related catalysts, including AuNP, ZrO_2_, etc. Compared with gold nanoparticles, they are cheaper and easier to obtain and may have great catalytic properties, which means that related application to Azo-STFs is worthy of further study to meet different environmental requirements and achieve controlled release and utilization of heat energy.

### Solvent

In addition, the influences of solvents, pH, and substituents of azobenzene derivatives on the thermal *cis*–*trans* isomerization cannot be ignored as well [155–157].

**Fig. 18:**
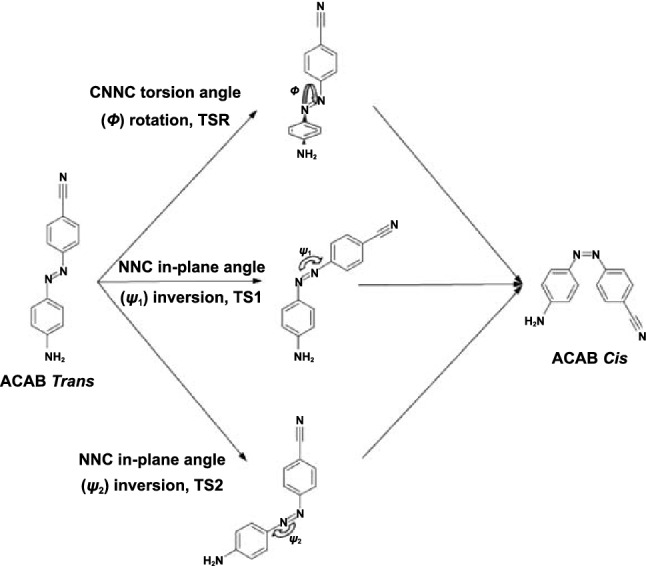
Mechanisms of *cis*–*trans* interconversion of 4-amino-4′-cyano-azobenzene [160]

As early as 1999, Gille [155] reported the rate constants of the thermal *cis*–*trans* isomerization of azobenzene dyes in solvents, acetone/water mixtures, and microheterogeneous surfactant solutions. His work showed that the effect of solvents on the photoisomerization of azobenzene is remarkable. The ground and low-lying excited free energy surfaces of 4-amino-4′-cyano azobenzene, a molecule that has been proposed as a building block for chiroptical switches, were studied by Corchado et al. [158] in gas phase and a variety of solvents (benzene, chloroform, acetone, and water). They used TD-DFT and CASPT2/CASSCF to analyze solvent effects on the absorption and emission spectra and on the *cis*–*trans* thermal and photoisomerization. They found that, in polar solvents, the mechanism of thermal *cis*–*trans* isomerization is controlled by the rotation of the C–N=N–C angle (Fig. 18). The change in the mechanism is mainly related to better solvation of the nitrogen atoms of the azo group in the rotational transition state. The photoisomerization follows a rotational pathway both in gas phase and solvents (polar and nonpolar). Solvents introduce only small modifications in the nπ* free energy surface (S1) but have a larger effect on the ππ* surface (S2), which in polar solvents gets closer to S1. The S2 band of the absorption spectrum is red-shifted by 0.27 eV for the *trans* isomer and by 0.17 eV for the *cis* isomer.

As the polarity of the solution affects the photoisomerization of azobenzene, this effect can be further expanded from the polarity to pH, etc. In 2014, Jurczak [159] found that the thermal *cis*/*trans* isomerization of simple azobenzene urea derivatives can be adjusted and controlled by anion binding, and the rate of this process strictly depends on the anion concentration and its binding force with the main *cis* isomer azobenzene. Jurczak attributed this phenomenon to the increase in the repulsive force between lone electron pairs in the N=N bond caused by the electron density transfer from the anion to the host *π*-system. Ochi [156] reported the effect of the structure of the phosphate groups (length and position), pH, and metal ions on the thermal *cis*–*trans* isomerization of water-soluble azobenzene derivatives. They revealed that the water-soluble azobenzene derivatives, in which di- or tri-phosphate group is substituted at the ortho position of the azobenzene via the amide linkage, undergo fast thermal isomerization at low pH. Zhang et al. [160] used DFT to systematically investigate the isomerization of neutral, cationic, and anionic azobenzene derivatives as well as the mechanism of the thermal reaction of various electron-donating and electron-withdrawing groups in the para position of the ground state benzene ring. The surface potential energy of radical anions/cations allows a significant reduction in the reaction energy barrier compared to the neutral state. The reaction energy barrier in thermally *cis* → *trans* isomerized anions and cations can be reduced by 18.8 and 24.2 kcal mol^−1^, respectively. The results showed that charge has a significant effect on the reaction mechanism. As the electron density distribution changes, the radical anion of the *cis* isomer can be rapidly isomerized to the corresponding *trans* configuration. In addition to the field of optical switches, this controllable electrochemical isomerization method can also be applied in the field of Azo-STFs. Owing to the overlapping *n*–π* transitions of the *trans* and *cis* isomers of azobenzene, it is actually difficult to obtain pure *trans* isomer in the Azo system, which also limits the power density and energy storage capacity of Azo-STFs. Therefore, Azo-compounds are always a mixture of *trans*- and *cis*-isomers in the photo-stable state [91, 161–165]. Yan et al. [166] found that the triggered self-assembly of *trans* isomers can promote the *cis*–*trans* transition if photostability is treated as a chemical equilibrium between *cis* and *trans* isomers. Their work also provided a meaningful research path for preparing high-efficiency Azo compounds by manipulating their *cis*-to-*trans* conversion.

While the pure solid Azo energy storage materials are the focus of mainstream current research, studies on the effects of solutions may still have high research significance for controlling the isomerization of azobenzene materials. The solutions can expand the use environment of Azo-STFs and significantly increase their flow capacity, which will be beneficial for us to replace energy storage materials after several cycles. In addition, compared with solid Azo-STFs, more functional substances can be carried in the solution, which is beneficial for us to further expand the application field of Azo-STFs.

### Electric Field and Magnetic Field

Interestingly, experiments have already provided evidence that the isomerization of azobenzene can be induced by resonant or inelastic tunneling of electrons [167].

Alemani et al. [147] chose 3,3′,5,5′-tetra-tert-butylazobenzene (TBA) to investigate the effect of directional electric field on isomerization. Their research has shown that the *trans*–*cis* isomerization of TBA molecules can be reversibly induced on Au(111). The relationship between the applied bias voltage necessary for switching and the tip height shows that the process is caused by the electric field between the STM tip and the sample surface.

**Fig. 19:**
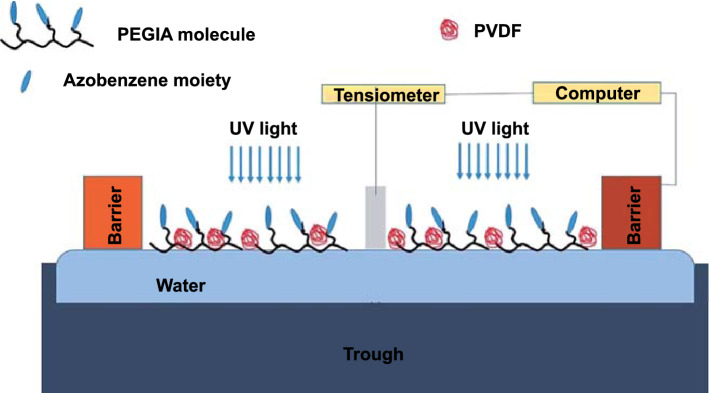
Mechanism of the effect of local electric field on *trans*–*cis* photoisomerization of azobenzene containing PEGIA [168]

The ability to control partial isomerization of azobenzene in the film using an electric field is very interesting for many nanoscale device applications. As the effect of directional external electric field on photoisomerization had already been studied, local electric field, generated by doping the film with polar molecules with a permanent dipole moment, could be used to adjust the rate of isomerization. Yogitha et al. [168] reported the effect of a local electric field on the rate of photoisomerization of azobenzene containing PEGIA-based side-chain liquid crystalline polymer in the ultra-thin film at the air–water interface (Fig. 19). The local electric field was realized by adding polar polyvinylidene difluoride (PVDF) molecules to the PEGIA thin film. The results showed the exponential effect of the percentage of PVDF, which can be attributed to the effect of the electric field owing to the dipole moment of the PVDF molecules. The effect of electric field on the isomerization of azobenzene demonstrated in the above study gives reasons to believe that the electric field can be used in combination with Azo-STFs under certain circumstances.

Electricity and magnetism do not exist separately. In 2014, Al-Aqtash et al. [169] reported the theoretical study of the light-induced switching of magnetization in core–shell nanoparticles. The core of the nanoparticle consists of Fe coated with the shell of azobenzene. This means that the magnetization of nanoparticles can be reversibly switched using specific wavelengths of light. Using an ab initio parameterization of magnetic interactions, Al-Aqtash et al. found that the magnetization of Fe nanoparticles can be switched by at least 50% owing to the photoisomerization of azobenzene at room temperature.

In 2021, Ji et al. [170] found that azobenzene-functionalized magnetic Fe_3_O_4_ nanoparticles can stabilize at liquid–liquid interface of polar N,N-dimethylformamide (DMF) and less polar toluene during photoisomerization, thereby forming dynamic emulsions controlled by light. Upon UV light irradiation, these Fe_3_O_4_ nanoparticles can be self-assembled into micrometer-sized aggregates. At the same time, polar DMF molecules are sequestered and harvested by Fe_3_O_4_ aggregates. Upon visible light irradiation, the subsequent release of these polar DMF molecules can also induce the secondary assembly of Fe_3_O_4_ nanoparticles at DMF/toluene interface, forming discrete DMF droplets stabilized by a monolayer of Fe_3_O_4_ nanoparticles.

As the isomerization of azobenzene can affect the formation of the magnetic field, the magnetic field in turn may also affect the isomerization of azobenzene under special condition, which may be worthy of further research. The variability of electric and magnetic fields makes the intelligent heat release of Azo-STFs possible. Combining azobenzene with magnetic fluids and other substances may be developed in the fields of tracking and soft robotics.

### Force

In 2012, Singleton et al. [171] investigated the photoinduced isomerization and thermal relaxation of an azobenzene-functionalized polymer poly(disperse red 1 acrylate) at increasing external pressures up to 1.5 GPa inside a diamond-anvil spectroscopic cell. Using laser pump–probe spectroscopy, they found that the isomerization half-life increases with pressure. It is worth noting that the pressure should not be excessive. Dong et al. [172] found that compression of 18 GPa results in an irreversible breakdown of the azobenzene molecular structure.

**Fig. 20:**
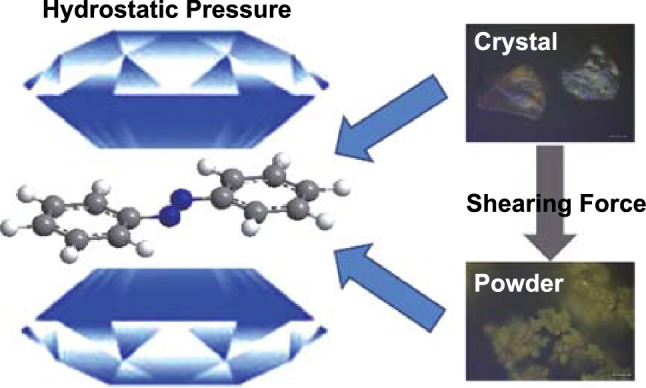
Structural changes in the *trans*-azobenzene single crystal and powder upon compression realized in the DAC [173]

Recently, Li et al. [173] reported the structural changes in the *trans*-azobenzene single crystal and powder upon compression using a diamond anvil cell (DAC) and Raman spectroscopy (Fig. 20). The shearing force destroyed the crystal structure of *trans*-azobenzene while the hydrostatic pressure caused *trans*-azobenzene to stack more closely and orderly. These results were proved by the blue shifts of Raman bands. During compression, the peaks at 1159, 1182, and 1593 cm^−1^, which are highly related to C–H in-plane bending, C–C stretching, and in-plane bending vibrations of the benzene ring, showed significant blue-shifts of Δ = 21, 35, and 21 cm^−1^, respectively. Li et al. attributed the different spectral behaviors of these two types of *trans*-azobenzene under hydrostatic pressure to the strong intermolecular interactions in the crystal form, which are different from those in the powder form. With the gradual decrease in pressure to the ambient pressure, the initial forms of the Raman spectra of *trans*-azobenzene were entirely recovered, indicating that the pressure-induced structural changes are reversible in a certain pressure range. This study further proved the effect of pressure on the isomerization of azobenzene.

In fact, pulling force can also affect the isomerization of azobenzene. Polymer films exhibit anisotropic photomechanical response if they have an oriented structure or are irradiated with linearly polarized light [174–176]. As early as 2008, Tanaka [177] used uniaxially stretched blend film composed of polycaprolactone and poly(vinyl ether) with azobenzene moiety (Azo-PVE) as a side chain to produce a rapid and reversible anisotropic photomechanical response in the polymer film. Moreover, the anisotropy of the photoresponse got more pronounced with the increase in the stretching ratio, reflecting the binding between amorphous areas through Azo-PVE long chains.

**Fig. 21:**
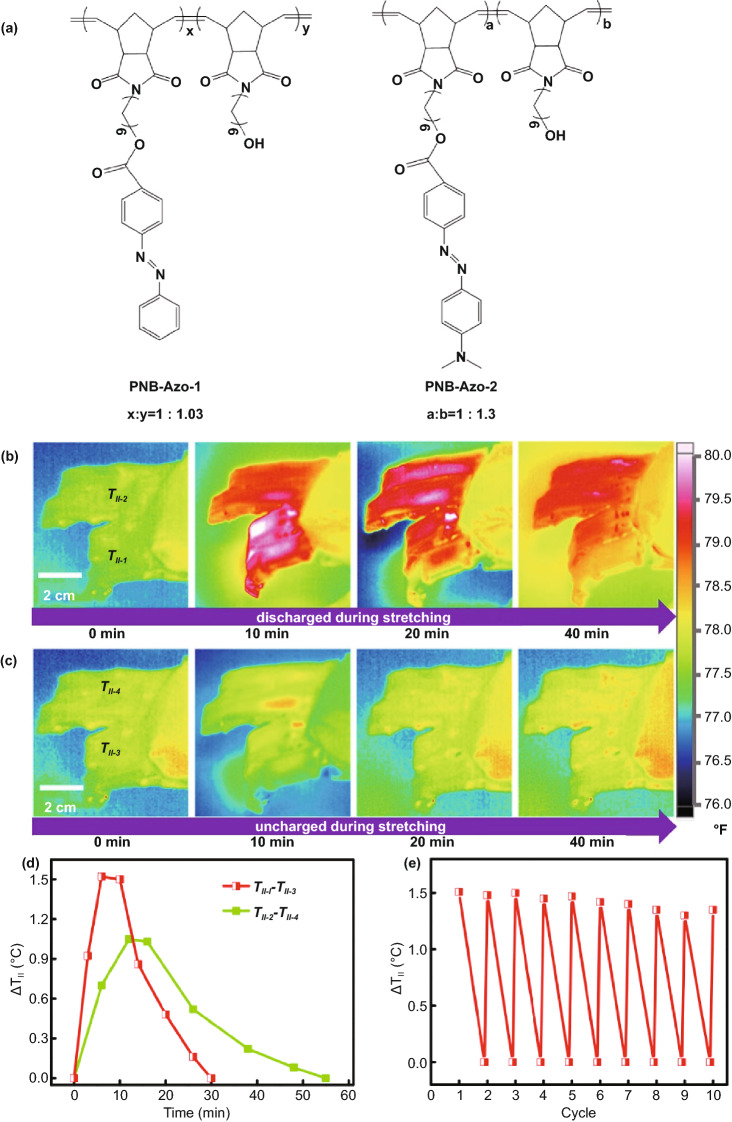
**a** Chemical structure of PNB-Azo-1 and PNB-Azo-2. **b** IR thermal images of charged four fingers (two fingers bend reversibly, II-1 and the other stay II-2). **c** IR thermal images of uncharged four fingers (two fingers bend reversibly, II-3 and the other stay II-4); **d** Δ*TII* at different times and **e** its change in 10 cycles. The temperature is obtained by averaging the data at 4 time points [178]

In fact, several studies have already been published on this topic. Feng et al. [178] prepared two azobenzene (PNB-Azo) materials with polynorbornene as a template through ring-opening metathesis polymerization and processed them into uniform flexible stretchable STF films (Fig. 21). The film had a high degree of isomerization and good storage stability. At a strain rate of 20%, the PNB-Azo film demonstrated high-intensity light charging (85%), high ED (49.0 Wh kg^−1^), high power density (161.0 W kg^−1^), and high-rate heat release caused by blue light (475 nm) irradiation at room temperature.

Feng used a high-resolution thermal imaging camera to track the temperature changes caused by the heat released under static and dynamic stretching during charging and discharging, and found that the reversible electric dynamic stretching during charging and discharging makes the PNB Azo “finger” release the heat faster than the statically stretched film, which can increase the temperature by 1.5 °C in a shorter period. The results showed that stretching can increase the solar thermal storage and release rate based on advantageous isomerization. This study also proved that it is feasible to use flexible stretchable PNB-Azo film for high-power dynamic solar heat sources by controlling deformation. Although there are few studies on deformation-controlled solar heat storage and release, this research direction is still worthy of further research. 

## Conclusions

In short, STFs based on azobenzene have been proven to be suitable and effective for solar thermal conversion and storage. In the current urgent need for clean energy, azobenzene has attracted increasing attention. At present, two aspects of molecular engineering and microstructure optimization, derivatives and template methods, are used to solve the problems of Azo-STFs: low ED, low quantum yield, short half-life, narrow overlap with the solar spectrum, and irreversible degradation.

**Fig. 22:**
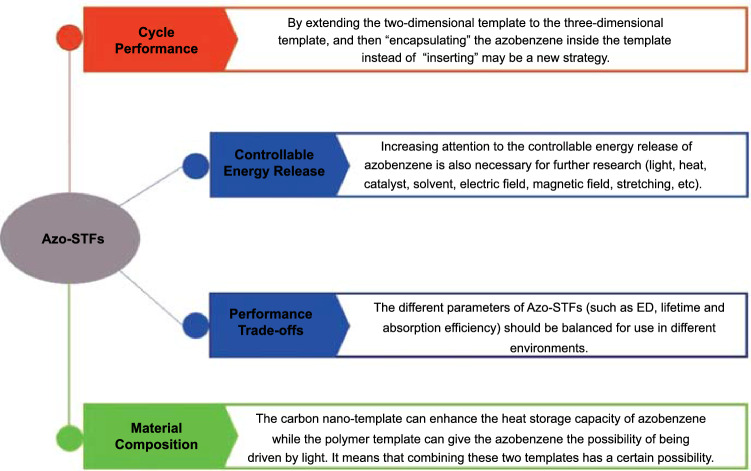
Current challenges and future prospects of Azo-STFs

Actually, azobenzene is still far from commercialization, but the biggest obstacle is not the energy storage density and half-life issues. In fact, some research results show that the energy storage efficiency of Azo-STFs is already comparable to that of lithium batteries. We believe that three main problems hinder the commercialization of Azo-STF. First, improvement in the performance of Azo-STFs in one aspect usually leads to a sacrifice in the performance in other aspects (Fig. 22). For example, the realization of full-spectrum Azo-STFs requires a large red shift of the absorption wavelength of the azobenzene optical switch, but this inevitably leads to poor ED and thermal stability; extending the half-life of Azo-STFs through substitution leads to a decrease in energy storage density. Therefore, different parameters of Azo-STFs (such as ED, lifetime, and absorption efficiency) should be balanced for use in different environments. Such adjustments cause the actual energy storage of the prepared Azo-STFs to fall short of theoretical expectations. Second, the control performance of Azo-STFs is poor. Most of the current research on Azo-STFs focuses on improving energy storage performance and extending half-life, and the energy storage performance values reported are only theoretical. As the photoisomerization of azobenzene is an equilibrium reaction, under the irradiation of artificial lasers with a short wavelength range, Azo-STFs exhibit incomplete isomerization even under artificial short-wavelength lasers. Under sunlight, which changes with the climate at any time, the results would be even more modest. In fact, many studies on the rational regulation of the photoisomerization of azobenzene, using metal nanoparticle catalysis and other methods, have been performed. Unfortunately, not many of the investigations combined energy storage research with research on regulating photoisomerization, and this field is worthy of further research. Finally, poor cycle performance of Azo-STFs is also their critical disadvantage. Azo-STFs templated with carbon nanomaterials can have cycling stability of hundreds of cycles, but that of Azo derivatives is only a dozen cycles. In addition, based on the energy storage mechanism of Azo-STFs, these materials are usually used in the form of thin films. Under sunlight and during exothermic cycling, thin films can be damaged irreversibly in a short period. Poor stability of the material also increases its use cost, to which, currently, there is no effective solution. Studies showed that because of the difference in *T*_*g*_ of *cis*- and *trans*-azobenzene derivatives, certain azobenzene materials can be used to repair damaged or cracked materials, which could be used to extend the service life of Azo-STFs films. The authors believe that the poor cycle performance of Azo energy storage system may be caused by its frequent isomerization during cycling. Although the theoretical recycling rate of the Azo unit is 100%, frequent isomerization may cause irreversible damage to the chemical bonds or the structure of the Azo unit. Template method, one of the most actively researched directions, consists of “inserting” the Azo unit into the carbon nano-template by grafting. Extremely high intermolecular forces in such materials can result in high energy storage capacity, comparable to that of commercial lithium batteries. However, if the Azo unit is damaged, there are no effective means of replacing it on the carbon nano-template. To this end, a strategy to achieve alternate mixing exotherm through MOFs material is proposed. It is true that replacing the two-dimensional structure with a 3D structure may damage the energy storage capacity of the Azo energy storage system to a certain extent, but as a potential way to effectively reduce its cost, this method may also be worthy of further development.

In addition to the storage of light and heat energy, azobenzene materials have also been applied in thermodynamic cycling in recent years. Azobenzene molecules undergo isomerization under light irradiation, causing a small change in molecular size (the molecular axis changes from 9 to 5.5 Å), which results in macroscopic visible deformation of the material. At present, it is already known that pulling force can promote the release of heat energy, implying a connection between azobenzene photothermal energy storage materials and azobenzene light-driven materials, which may become a future research prospect in azobenzene materials.

Although the field of Azo-STFs is in its infancy, Azo-STFs provide a new way for solar energy collection and storage. Future research will focus on attaining high ED, long storage life, controllable heat release, and excellent cycling stability. Azobenzene-related materials have great potential and therefore are expected to be widely applied in the field of solar energy collection and storage such as deicing, desalination, and heat-driven generators in the current era of high sustainable energy.
